# Diffuse gliomas classified by 1p/19q co-deletion, *TERT* promoter and IDH mutation status are associated with specific genetic risk loci

**DOI:** 10.1007/s00401-018-1825-z

**Published:** 2018-02-19

**Authors:** Karim Labreche, Ben Kinnersley, Giulia Berzero, Anna Luisa Di Stefano, Amithys Rahimian, Ines Detrait, Yannick Marie, Benjamin Grenier-Boley, Khe Hoang-Xuan, Jean-Yves Delattre, Ahmed Idbaih, Richard S. Houlston, Marc Sanson

**Affiliations:** 1 Sorbonne Universités UPMC Univ Paris 06, INSERM CNRS, U1127, UMR 7225, ICM, 75013 Paris, France; 2 Division of Genetics and Epidemiology, The Institute of Cancer Research, Sutton, Surrey SM2 5NG UK; 3 Service de neurologie 2-Mazarin, AP-HP, Groupe Hospitalier Pitié-Salpêtrière, Paris, France; 4 University of Pavia and C. Mondino National Institute of Neurology, Pavia, Italy; 5 Univ. Lille, Inserm, Institut Pasteur de Lille, U1167-RID-AGE-Risk Factors and Molecular Determinants of Aging-Related Diseases, 59000 Lille, France

## Abstract

Recent genome-wide association studies of glioma have led to the discovery of single nucleotide polymorphisms (SNPs) at 25 loci influencing risk. Gliomas are heterogeneous, hence to investigate the relationship between risk SNPs and glioma subtype we analysed 1659 tumours profiled for IDH mutation, *TERT* promoter mutation and 1p/19q co-deletion. These data allowed definition of five molecular subgroups of glioma: triple-positive (IDH mutated, 1p/19q co-deletion, *TERT* promoter mutated); *TERT*-IDH (IDH mutated, *TERT* promoter mutated, 1p/19q-wild-type); IDH-only (IDH mutated, 1p/19q wild-type, *TERT* promoter wild-type); triple-negative (IDH wild-type, 1p/19q wild-type, *TERT* promoter wild-type) and *TERT*-only (*TERT* promoter mutated, IDH wild-type, 1p/19q wild-type). Most glioma risk loci showed subtype specificity: (1) the 8q24.21 SNP for triple-positive glioma; (2) 5p15.33, 9p21.3, 17p13.1 and 20q13.33 SNPs for *TERT*-only glioma; (3) 1q44, 2q33.3, 3p14.1, 11q21, 11q23.3, 14q12, and 15q24.2 SNPs for IDH mutated glioma. To link risk SNPs to target candidate genes we analysed Hi-C and gene expression data, highlighting the potential role of *IDH1* at 2q33.3, *MYC* at 8q24.21 and *STMN3* at 20q13.33. Our observations provide further insight into the nature of susceptibility to glioma.

**Electronic supplementary material:**

The online version of this article (10.1007/s00401-018-1825-z) contains supplementary material, which is available to authorized users.

## Introduction

Diffuse gliomas are the most common malignant primary brain tumour affecting adults with around 26,000 newly diagnosed cases each year in Europe [9]. Diffuse gliomas have traditionally been classified into oligodendroglial and astrocytic tumours and are graded II–IV, with the most common form—Glioblastoma (GBM) or glioma grade IV—typically having a median survival of only 15 months [2].

Despite glioma being an especially devastating malignancy little is known about its aetiology and aside from exposure to ionising radiation that accounts for very few cases no environmental or lifestyle factor has been unambiguously linked to risk [2]. Recent genome-wide association studies (GWAS) have, however, enlightened our understanding of glioma genetics identifying single-nucleotide polymorphisms (SNPs) at multiple independent loci influencing risk [22, 25, 35, 44, 49, 51, 63]. While understanding the functional basis of these risk loci offers the prospect of gaining insight into the development of glioma, few have been deciphered. Notable exceptions are the 17p13.1 locus, where the risk SNP rs78378222 disrupts *TP53* polyadenylation [51] and the 5p15.33 locus, where the risk SNP rs10069690 creates a splice-donor site leading to an alternate *TERT* splice isoform lacking telomerase activity [24].

Since the aetiological basis of glioma subtypes is likely to reflect different developmental pathways it is not perhaps surprising that subtype-specific associations have been shown for GBM (5p15.33, 7p11.2, 9p21.3, 11q14.1, 16p13.33, 16q12.1, 20q13.33 and 22q13.1) and for non-GBM glioma (1q44, 2q33.3, 3p14.1, 8q24.21, 10q25.2, 11q21, 11q23.2, 11q23.3, 12q21.2, 14q12 and 15q24.2) [35]. Recent large-scale sequencing projects have identified IDH mutation, *TERT* promoter mutation and 1p/19q co-deletion as cancer drivers in glioma. These findings have improved the subtyping of glioma [5, 12, 26, 27] and this information has been incorporated into the revised 2016 WHO classification of glial tumours [32]. Since these mutations are early events in glioma development, any relationship between risk SNP and molecular profile should provide insight into glial oncogenesis. Evidence for the existence of such subtype specificity is already provided by the association of the 8q24.21 (rs55705857) risk variant with 1p/19q co-deletion, IDH mutated glioma [13]. Additionally, it has been proposed that associations may exist between risk SNPs at 5p15.33, 9p21.3 and 20q13.33 and IDH wild-type glioma [10], as well as 17p13.1 and *TERT* promoter, IDH mutated glioma without 1p/19q co-deletion [12].

To gain a more comprehensive understanding of the relationship between the 25 glioma risk loci and tumour subtype we analysed three patient series totalling 2648 cases. Since generically the functional basis of GWAS cancer risk loci appear primarily to be through regulatory effects [53], we analysed Hi-C and gene expression data to gain insight into the likely target gene/s of glioma risk SNPs.

## Materials and methods

### Data sources

We analysed data from three non-overlapping case series: TCGA, French GWAS, French sequencing. Details of these datasets are provided below and are summarised in Table 1.

**Table 1:** Overview of TCGA, French GWAS and French seq series and mutation status of tumours

Dataset	Controls	Cases (GBM/non-GBM)	Case groupings																	
			IDH status		*EGFR*		*CDKN2A*		Molecular subgroup						WHO 2016 classification					
			mut	wt	amp	wt	del	wt	IDH-only	*TERT*-IDH	*TERT*-only	Triple −ve	Triple +ve	Total	AstroIDH-mut	Astro IDH-wt	Oligo 1p19q	GBM IDH-mut	GBM IDH-wt	Total
TCGA	2648	521 (183/338)	293	228	246	270	254	262	100	4	45	10	65	224	166	51	116	10	171	514
French GWAS	1190	1423 (430/993)	366	498	118	628	173	573	169	46	309	141	85	750	188	214	95	27	233	757
French seq	5527	704 (181/523)	427	277	101	592	144	549	181	28	185	92	199	685	178	114	218	31	148	689
Total	9365	2648 (795/1854)	1086	1003	465	1490	571	1384	450	78	539	243	349	1659	532	379	429	68	552	1960

*Amp* amplified, *astro* astrocytoma, *del* deleted, *mut* mutated, *oligo* oligodendroglioma, *wt* wildtype

### TCGA

Raw genotyping files (.CEL) for the Affymetrix Genome-wide version 6 array were downloaded for germline (i.e. normal blood) glioma samples from The Cancer Genome Atlas (TCGA, dbGaP study accession: phs000178.v1.p1). Controls were from publicly accessible genotype data generated by the Wellcome Trust Case–Control Consortium 2 (WTCCC2) analysis of 2699 individuals from the 1958 British birth cohort (1958-BC) [41]. Genotypes were generated using the Affymetrix Power Tools Release 1.20.5 using the Birdseed (v2) calling algorithm (https://www.affymetrix.com/support/developer/powertools/changelog/index.html) and PennCNV [59]. After quality control (Supplementary Figs. 1, 2, Supplementary Table 1) there were 521 TCGA glioma cases and 2648 controls (Table 1). Glioma tumour molecular data (IDH mutation, 1p/19q co-deletion, *TERT* promoter mutation) were obtained from Ceccarelli et al. [6]. Further data (*EGFR* amplification/activating mutations, *CDKN2A* deletion) were obtained from the cBioportal for cancer genomics [15]. After adjustment for principal components there was minimal evidence of over-dispersion inflation (*λ* = 1.01; Supplementary Fig. 2).

### French GWAS

The French-GWAS [25, 44] comprised 1423 patients with newly diagnosed grade II–IV diffuse glioma attending the Service de Neurologie Mazarin, Groupe Hospitalier Pitié-Salpêtrière Paris. The controls (*n* = 1190) were ascertained from the SU.VI.MAX (SUpplementation en VItamines et MinerauxAntioXydants) study of 12,735 healthy subjects (women aged 35–60 years; men aged 45–60 years) [19]. Tumours from patients were snap-frozen in liquid nitrogen and DNA was extracted using the QIAmp DNA minikit, according to the manufacturer’s instructions (Qiagen, Venlo, LN, USA). DNA was analysed for large-scale copy number variation by comparative genomic hybridisation (CGH) array as previously described [16, 21]. For tumours not analysed by CGH array, 1p/19q co-deletion status was assigned using PCR microsatellites, and *EGFR*-amplification and *CDKN2A*-*p16*-*INK4a* homozygous deletion by quantitative PCR. *IDH1*, *IDH2* and *TERT* promoter mutation status was assigned by sequencing [26, 45].

### French sequencing

Eight hundred and fifteen patients newly diagnosed grade II–IV diffuse glioma were ascertained through the Service de Neurologie Mazarin, Groupe Hospitalier Pitié-Salpêtrière Paris. Genotypes for the 25 risk SNPs were obtained by universal-tailed amplicon sequencing in conjunction with Miseq technology (Illumina Inc.). Genotypes were called using GATK (Genome Analysis ToolKit, version 3.6-0-g89b7209) software. Duplicated samples and individuals with low call rate (< 90%) were excluded (*n* = 111). Molecular profiling of tumour samples was carried out as per the French GWAS.

Unrelated French controls were obtained from the 3C Study (Group 2003) [17] a population-based, prospective study of the relationship between vascular factors and dementia being carried out in Bordeaux, Montpellier, and Dijon. Genotyping of controls was performed using Illumina Human 610-Quad BeadChips. To recover untyped genotypes imputation using IMPUTE2 software was performed using 1000 genomes multi-ethnic data (1000 G phase 1 integrated variant set release v3) as reference. SNPs genotypes were retained call rates were > 98%, Hardy–Weinberg equilibrium (HWE) *P* value > 1 × 10^−6^, minor allele frequency (MAF) > 1%. After quality control, 704 cases and 5527 controls were available for analysis (Table 1).

### Statistical analysis

Test of association between SNP and glioma molecular subgroup was performed using SNPTESTv2.5 [33] under an additive frequentist model. Where appropriate, principal components, generated using common SNPs, were included in the analysis to limit the effects of cryptic population stratification that otherwise might cause inflation of test statistics. Eigenvectors for the TCGA study were inferred using smartpca (part of EIGENSOFTv2.4) [40] by merging cases and controls with phase II HapMap samples [25].

To ensure reliability when restricting cases to per-group low sample counts, imputed genotypes were thresholded at a probability > 0.9 (e.g. –method threshold in SNPtest) for the TCGA and French-GWAS studies. For the French-sequence study we used –method expected, as we were comparing genotypes from directly sequenced cases against imputed controls. We compared control frequencies to those from European 1000 genomes project to ensure the validity of this approach.

Meta-analyses were performed using the fixed-effects inverse-variance method based on the β estimates and standard errors from each study using META v1.6 [30]. Cochran’s *Q* statistic was used to test for heterogeneity [20].

### Risk allele number and age at diagnosis

For imputed SNPs a genotype probability threshold > 0.9 was used. The age and survival distribution of cases carrying additive combinations of risk alleles were assessed for the 25 SNPs across the molecular subgroups. Trend lines were estimated using linear regression in *R* and plotted using the *ggplot2* package [62]. Association between risk allele number and age was assessed using Pearson correlation.

### Survival analysis

Survival plots were generated using the *survfit* package in *R* which computes an estimate of a survival curve for censored data using the Kaplan–Meier method. Log-rank tests were used to compare curves between groups and power to demonstrate a relationship between different groups and overall survival was estimated using sample size formulae for comparative binomial trials. The Cox proportional-hazards regression model was used to investigate the association between survival and age, grade, molecular group and number of risk alleles. Individuals were excluded if they died within a month of surgery. Date of surgery was used as a proxy for the date of diagnosis.

### Expression quantitative trait locus analysis

We searched for expression quantitative trait loci (eQTLs) in 10 brain regions using the V6p GTEx [31] portal (https://gtexportal.org/home/) as well as in whole blood using the blood eQTL browser [61] (https://molgenis58.target.rug.nl/bloodeqtlbrowser/).

### Hi-C analysis

We examined for significant contacts between glioma risk SNPs and nearby genes using the HUGIn browser [34], which is based on analysis by Schmitt et al. [48]. We restricted the analysis to Hi-C data generated on H1 Embryonic Stem Cell and Neuronal Progenitor cell lines, as originally described in Dixon et al. [11]. Plotted topologically associating domain (TAD) boundaries were obtained from the insulating score method [8] at 40-kb bin resolution. We searched for significant interactions between bins overlapping the glioma risk SNP and all other bins within 1 Mb at each locus (i.e. “virtual 4C”).

### Gene set enrichment analysis

Gene set enrichment analysis (GSEA) was carried out using version 3.0 with gene sets from Molecular Signatures Database (MSigDB) v6.0 [36, 52], restricted to the C2 canonical pathways sets (*n* = 1329). Analysis was carried out using default settings, with the exception of removing restrictions on gene set size. RSEM normalised mRNASeq expression data for 20,501 genes in 676 glioma cases from TCGA were downloaded from the Broad Institute TCGA GDAC (http://gdac.broadinstitute.org/). These were assigned molecular groupings using sample information from Supplementary Table 1 of Ceccarelli et al. [6].

## Results

### Descriptive characteristics of datasets

**Fig. 1:**
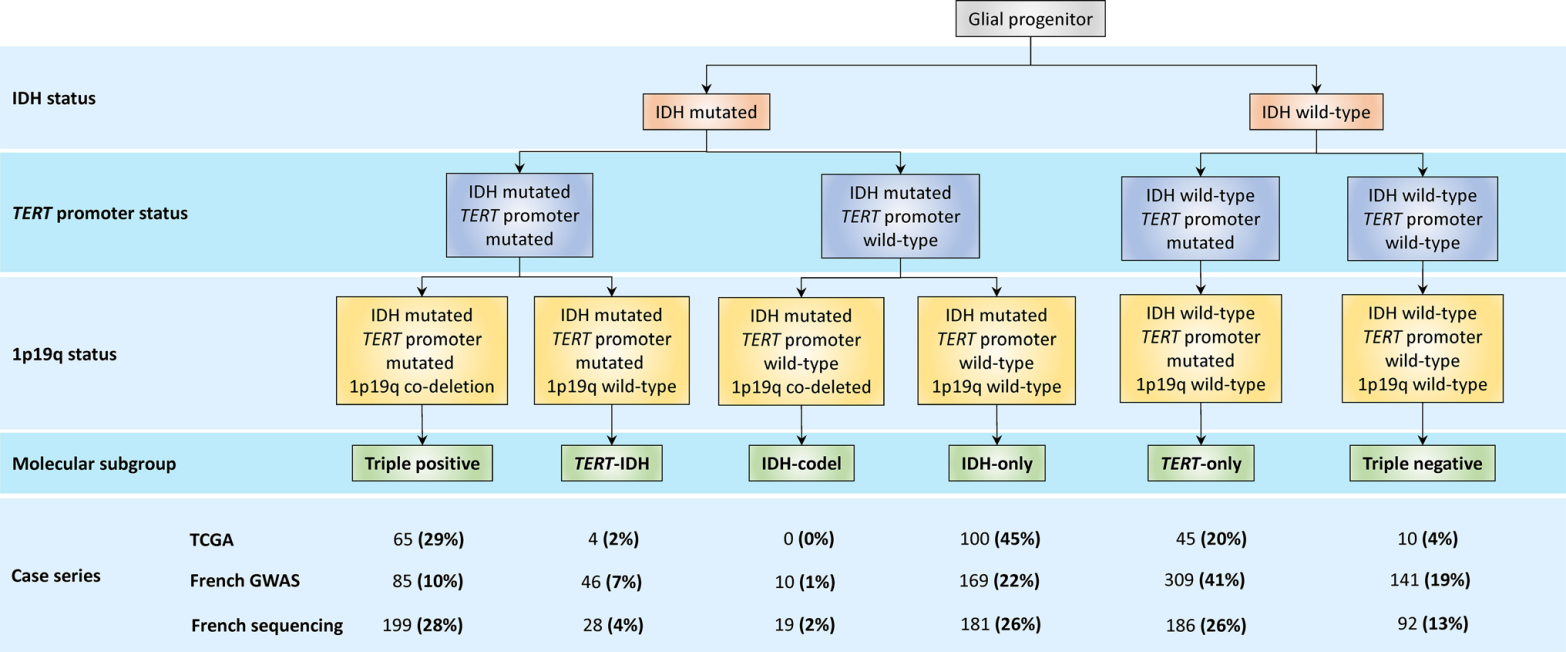
Molecular classification of diffuse glioma and frequency of each subgroup in the TCGA, French-GWAS and French sequencing case series

We studied three non-overlapping glioma case–control series of Northern European ancestry totalling 2648 cases and 9365 controls (Table 1). For 1659 of the 2648 cases information on tumour, 1p/19q co-deletion, *TERT* promoter and IDH mutation status was available (Fig. 1). Using these data allowed definition of five molecular subgroups of glioma: triple-positive (IDH mutated, 1p/19q co-deletion, *TERT* promoter mutated); *TERT*-IDH (IDH mutated, *TERT* promoter mutated, 1p/19q-wild-type); IDH-only (IDH mutated, 1p/19q wild-type, *TERT* promoter wild-type); *TERT*-only (*TERT* promoter mutated, IDH wild-type, 1p/19q wild-type) and triple-negative (IDH wild-type, 1p/19q wild-type, *TERT* promoter wild-type). As only 29 cases were classified as IDH mutation, 1p/19q co-deletion and *TERT* promoter wild-type, we restricted subsequent analyses to the five groups as above. Table 1 also shows grouping of the 1960 cases adopting the WHO 2016 classification of glial tumours into five categories (Astrocytoma with IDH mutation, IDH wild-type astrocytoma, Oligodendroglioma with 1p/19q co-deletion, GBM with IDH mutation and IDH wild-type GBM) (Supplementary Table 2 [Online Resource 1]).

### SNP selection

We analysed 25 SNPs, which had been reported to show the strongest genome-wide significant association with glioma in our recent meta-analysis of 12,496 cases and 18,190 controls [35] (Table 2). In the current analysis all of the SNPs exhibited a consistent direction of effect with that previously reported, albeit some weakly [Supplementary Fig. 4 (Online Resource 1), Supplementary Table 3 (Online Resource 2)].

**Table 2:** Overview of glioma risk SNPs at the 25 loci

Locus	SNP	Alleles	RAF	Reported subtype
1p31.3 [35]	rs12752552 [35]	**T**/C	0.87	GBM
1q32.1 [35]	rs4252707 [35]	G/**A**	0.22	Non-GBM
1q44 [35]	rs12076373 [35]	**G**/C	0.84	Non-GBM
2q33.3 [35]	rs7572263 [35]	**A**/G	0.76	Non-GBM
3p14.1 [35]	rs11706832 [35]	A/**C**	0.46	Non-GBM
5p15.33 [49]	rs10069690 [35]	C/**T**	0.28	GBM
7p11.2 [44]	rs75061358 [35]	T/**G**	0.10	GBM
7p11.2 [44]	rs11979158 [44]	**A**/G	0.83	GBM
8q24.21 [49]	rs55705857 [13, 22]	A/**G**	0.06	Non-GBM
9p21.3 [49, 63]	rs634537 [35]	T/**G**	0.41	GBM
10q24.33 [35]	rs11598018 [35]	**C**/A	0.46	Non-GBM
10q25.2 [25]	rs11196067 [25]	A/**T**	0.58	Non-GBM
11q14.1 [35]	rs11233250 [35]	**C**/T	0.87	GBM
11q21 [35]	rs7107785 [35]	**T**/C	0.48	Non-GBM
11q23.2 [25]	rs648044 [25]	**A**/G	0.39	Non-GBM
11q23.3 [49]	rs12803321 [35]	**G**/C	0.64	Non-GBM
12q21.2 [25]	rs1275600 [35]	**T**/A	0.60	Non-GBM
14q12 [35]	rs10131032 [35]	**G**/A	0.92	Non-GBM
15q24.2 [25]	rs77633900 [35]	G/**C**	0.09	Non-GBM
16p13.3 [35]	rs2562152 [35]	A/**T**	0.85	GBM
16p13.3 [35]	rs3751667 [35]	C/**T**	0.21	Non-GBM
16q12.1 [35]	rs10852606 [35]	T/**C**	0.71	GBM
17p13.1 [51]	rs78378222 [51]	T/**G**	0.01	All
20q13.33 [49, 63]	rs2297440 [35]	T/**C**	0.80	GBM
22q13.1 [35]	rs2235573 [35]	**G**/A	0.51	GBM

The risk allele frequency (RAF) is from European samples from 1000 genomes project. At 10q25.2, rs11599775 [35] failed sequencing so the originally reported SNP rs11196067 [25] was used

The risk allele is emboldened and the minor allele underlined

### Relationship between risk SNP and molecular subgroup

In the first instance, we examined whether the associations at the 25 risk loci were broadly defined by IDH status. We observed significant association for IDH mutated group with 1q44 (rs12076373), 2q33.3 (rs7572263), 3p14.1 (rs11706832), 8q24.21 (rs55705857), 11q21 (rs7107785), 11q23.3 (rs12803321), 14q12 (rs10131032), 15q24.2 (rs77633900) and 17p13.1 (rs78378222) risk SNPs. In addition, we found strong associations with IDH wild-type gliomas at 5p15.33 (rs10069690), 7p11.2 (rs75061358), 9p21.3 (rs634537), and 20q13.33 (rs2297440) (Supplementary Fig. 5 [Online Resource 1], Supplementary Table 3 [Online Resource 2]). Of particular note was the finding that many of the risk loci recently discovered which were reported to be associated with non-GBM (1q44, 2q33.3, 3p14.1, 11q21, 14q12, 15q24.2) [35] showed a strong association with IDH mutant glioma.

**Fig. 2:**
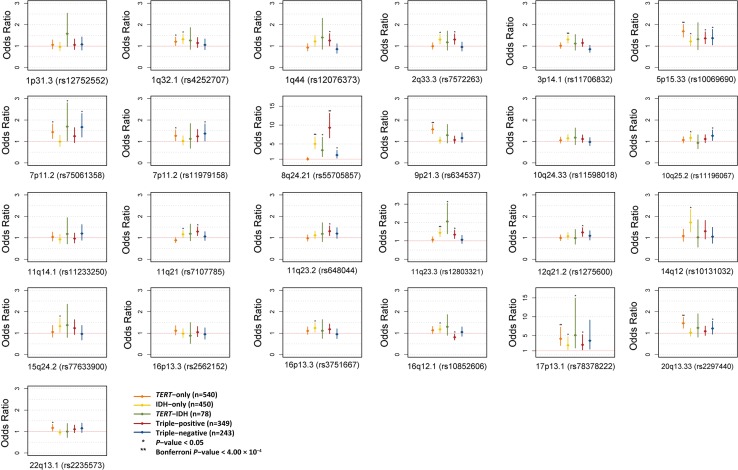
Association between the 25 risk loci and glioma subgroup. Horizontal red line corresponds to an odds ratio of 1.0

Following on from this we performed a more detailed stratified analysis based on classifying the glioma tumours into the five molecularly defined groups. We found a strong association with IDH mutated tumours at 8q24.21 (rs55705857), in particular with triple-positive glioma [*P* = 1.27 × 10^−37^, OR = 9.30 (6.61–13.08)], which corresponds to the WHO 2016 oligodendroglioma classification [Supplementary Fig. 6 (Online Resource 1), Supplementary Table 3 (Online Resource 2)]. Furthermore, we confirmed the previously reported associations at 5p15.33 (rs10069690), 9p21.3 (rs634537), 17p13.1 (rs78378222) and 20q13.33 (rs2297440) with *TERT*-only glioma in each of the three series [12]. Finally, we found suggestive evidence for an association between 22q13.1 (rs2235573) with *TERT*-only glioma, as well as 11q21 (rs7107785), 11q23.2 (rs648044), and 12q21.2 (rs1275600) with triple-positive glioma [Fig. 2, Supplementary Table 3 (Online Resource 2)].

In addition to data on 1p/19q co-deletion, *TERT* promoter and IDH mutation, for 1955 of the tumours we had information on *EGFR* amplification and *CDKN2A* deletion status (Table 1). Using these data we examined for an association with *EGFR* amplification and *CDKN2A* deletion, particularly focusing on the 7p11.2 (rs75061358 and rs11979158) and 9p21.3 (rs634537) risk SNPs in view of the fact that these loci map in or near *EGFR* and *CDKN2A,* respectively (Supplementary Figs. 7, 8 [Online Resource 1], Supplementary Table 3 [Online Resource 2]). At 7p11.2, the intergenic variant rs75061358, which is located in the genomic vicinity of *EGFR,* was associated with *EGFR* amplified tumours and not those without amplification. There was a less strong association with EGFR amplification seen with the second independent signal at the locus defined by rs11979158, which is intronic within *EGFR* itself. At 9p21.3 rs634537, which is intronic within *CDKN2B*-*AS1* and in the vicinity of *CDKN2A* and *CDKN2B*, was not associated with *CDKN2A* deletion status. Low grade gliomas tend to be *EGFR* wild-type and *p16* wild-type tumours and, therefore, as anticipated many non-GBM risk SNPs were most strongly associated with these tumours; notably 2q33.3 (rs7572263), 3p14.1 (rs11706832), 8q24.21 (rs55705857), 10q25.2 (rs11196067), 11q23.3 (rs12803321) (Supplementary Figs. 7, 8 [Online Resource 1], Supplementary Table 3 [Online Resource 2]).

### Polygenic contribution to age at diagnosis and patient survival

Patient survival by molecular subgroup in each of the three series was consistent with previous published reports [5, 12]; specifically, patients with triple-positive tumours had the best prognosis whilst those with *TERT*-only tumours had the worst outcome (Supplementary Fig. 3 [Online Resource 1]). We investigated whether an increased burden of glioma risk alleles might be associated with earlier age at diagnosis (i.e. indicative of influence on glioma initiation) or survival (indicative of influence on glioma progression). There was a slight albeit, non-significant trend towards decreased age at diagnosis with increased risk allele number in the IDH-only, *TERT*-only and triple-positive molecular subgroup, but with decreased risk allele number in the *TERT*-IDH and Triple-negative tumours (Supplementary Fig. 9 [Online Resource 1]). We found no overall relationship between age and risk allele number, or for the individual molecular groups (Supplementary Table 4 [Online Resource 1]). Examining each SNP individually, only rs55705857 at 8q24.21 was nominally associated with age (Supplementary Table 4 [Online Resource 1]).

We used Cox Proportional-Hazards Regression to investigate whether burden of glioma risk was associated with survival, with each risk allele coded as 0, 1 or 2. As expected, age, grade and all molecular group (Triple-negative, Triple-positive, *TERT*-only, IDH-only and *TERT*-IDH) were strongly associated with decreased survival. Intriguingly, the number of risk alleles was associated with increased survival (Supplementary Table 5 [Online Resource 1]; *P* < 10^−4^) with 1q32.1 (rs4252707), 11q23.3 (rs12803321) and 11q21 (rs7107785) each being nominally associated with survival, independent of age and molecular subgroup. Considering the relationship between burden of glioma risk alleles and survival in each molecular subgroup a consistent association with increased survival was shown in Triple-positive, Triple-negative and *TERT*-only molecular groups but not in IDH-only and *TERT*-IDH groups.

### Biological inference of risk loci

Since genomic spatial proximity and chromatin looping interactions are fundamental for the regulation of gene expression [42], we interrogated physical interactions at respective risk loci in embryonic stem cells and neuronal progenitor cells using Hi-C data. We also sought to gain insight into the possible biological mechanisms for associations by performing expression quantitative trait locus (eQTL) analysis using mRNA expression data in 10 brain regions using the GTEx portal.

We identified significant Hi-C contacts from the genomic regions which encompass 14 of the 25 risk loci implicating a number of presumptive candidate genes. For two of these, candidacy was supported by eQTL data. (Table 3; Supplementary Fig. 10 [Online Resource 1]; Supplementary Table 6 [Online Resource 3]). Notably at 2q33.3, there was a significant looping interaction between the risk SNP and *IDH1*/*IDH1*-*AS1*, as well as with *EGFR/EGFR*-*AS1* at 7p11.2, *CDKN2A/CDKN2B* at 9p21.3, *NFASC* at 1q32.1 and *LRIG1* at 3p14.1. At the 8q24.21 gene desert Hi-C data revealed a significant interaction between the risk SNP rs55705857 and *MYC*, as well as lincRNAs in the region such as *PCAT1/PCAT2*. Additionally, the risk SNP rs12803321 at 11q23.3 was significantly associated with *PHLDB1* expression in the brain.

**Table 3:** Candidate gene basis of glioma risk loci

Locus	SNP	Molecular group	IDH, *EGFR*, *CDKN2A* status	eQTL (tissue)/Hi-C	Commentary
1p31.3	rs12752552	–	–	*JAK1* (brain)/*RAVER2, JAK1, UBE2U, CACHD1*	JAK1 is involved in actomyosin contractility in tumour cells and stroma to aid metastasis [46]
1q32.1	rs4252707	*TERT*-only*, IDH-only*	IDHmut*, *EGFR*wt*, *CDKN2A*wt*	*NFASC*	NFASC is a cell adhesion molecule involved in axon subcellular targeting and synapse formation during neural development [1]
1q44	rs12076373	TP*	IDHmut**	*AKT3, ZBTB18, SDCCAG8*	AKT3 is highly expressed in brain, regulates cell signalling in response to insulin and growth factors [4], involved in regulation of normal brain size [28]
2q33.3	rs7572263	IDH-only*, TP*	IDHmut**, *EGFR*wt*, *CDKN2A*wt*	*IDH1, IDH1*-*AS1*	IDH mutant protein overexpression increases glioma cell radiation sensitivity [29]
3p14.1	rs11706832	IDH-only**	IDHmut**, EGFRwt*, *CDKN2A*wt*	*LRIG1* (blood), *SLC25A26* (blood)/*LRIG1*	–
5p15.33	rs10069690	*TERT*-only**, IDH-only*, TP*, TN*	IDHmut*, IDHwt**, *EGFR*amp**, *EGFR*wt*, *CDKN2A*del*, *CDKN2A*wt**	–	rs10069690 affects *TERT* splicing [24]
7p11.2	rs75061358	*TERT*-only*, *TERT*-IDH*, TN*	IDHwt**, *EGFR*amp**, *CDKN2A*wt*	–	–
7p11.2	rs11979158	*TERT*-only*, TN*	IDHwt*, *EGFR*amp*, *EGFR*wt*, *CDKN2A*del*, *CDKN2A*wt*	*EGFR, EGFR*-*AS1*	–
8q24.21	rs55705857	IDH-only**, *TERT*-IDH*, TP**, TN*	IDHmut**, *EGFR*wt*, *CDKN2A*wt**, *CDKN2A*del**	*PCAT1, PCAT2, CASC8, CASC11, MYC, PVT1*	–
9p21.3	rs634537	*TERT*-only**	IDHwt**, *EGFR*amp*, *EGFR*wt*, *CDKN2A*del*, *CDKN2A*wt**	*CDKN2A, CDKN2B*-*AS1*	–
10q24.33	rs11598018	–	IDHmut*, *EGFR*wt*	*GSTO1, GSTO2* *SH3PXD2A*	Correlated SNP to rs11598018 associated with telomere length likely through *OBFC1* [7]
10q25.2	rs11196067	IDH-only*, TN*	IDHmut*, IDHwt*, *EGFR*wt*, *CDKN2A*wt*	*TCF7L2, VTI1A, HABP2*	TCF7L2 modifies beta-catenin signalling and controls oligodendrocyte differentiation [69]
11q14.1	rs11233250	–	–	–	–
11q21	rs7107785	IDH-only*, TP*	IDHmut**, *EGFR*wt*, *CDKN2A*del*	*RP11*-*712B9.2* (brain)	–
11q23.2	rs648044	TP*	IDHmut*, *EGFR*wt**, *CDKN2A*wt**	*NNMT, ZBTB16*	NNMT is upregulated in GBM, NAD metabolism important in glioma [23]
11q23.3	rs12803321	IDH-only**, *TERT*-IDH*, TP*	IDHmut**, *EGFR*wt**, *CDKN2A*wt**, *CDKN2A*del*	*PHLDB1* (brain)	PHLDB1 is an insulin-responsive protein that enhances Akt activation [70]
12q21.2	rs1275600	TP*	IDHmut*, *EGFR*wt**, *CDKN2A*wt**, *CDKN2A*del*	*KRR1, GLIPR1*	GLIPR1 is targeted by TP53 [43]
14q12	rs10131032	IDH-only*	IDHmut**, *EGFR*wt*, *CDKN2A*del*, *CDKN2A*wt*	*NPAS3*	NPAS3 is a tumour suppressor for astrocytoma [37]
15q24.2	rs77633900	IDH-only*	IDHmut**, *EGFR*wt*, *CDKN2A*wt*	*SCAPER*	–
16p13.3	rs2562152	–	–	–	–
16p13.3	rs3751667	IDH-only*	IDHmut*, *EGFR*amp*, *EGFR*wt*, *CDKN2A*wt*	*RP11*-*161M6.2* (brain), *SOX8* (blood)	SOX8 is strongly expressed in brain and may be involved in neural development [47]
16q12.1	rs10852606	IDH-only*, TP* (−ve)	–	*HEATR3* (brain)	HEATR3 may be involved in NOD2-mediated NF-kappa B signalling [67]
17p13.1	rs78378222	*TERT*-only**, IDH-only*, *TERT*-IDH*, TP*	IDHmut**, IDHwt*, *EGFR*amp*, *EGFR*wt*, *CKDN2A*wt**, *CDKN2A*del*	–	rs78378222 affects *TP53* 3′UTR poly-adenylation processing [51]
20q13.33	rs2297440	*TERT*-only**, TN*	IDHwt**, *EGFR*amp**, *EGFR*wt*, *CDKN2A*del*, *CDKN2A*wt*	*STMN3* (brain)*, LIME1* (blood)*, ZGPAT* (blood)*, EEF1A2* (blood)	Overexpression of STMN3 promotes growth in GBM cells [68]
22q13.1	rs2235573	*TERT*-only*	IDHwt*	*CTA*-*228A9.3* (brain)	–

*TN* triple negative (i.e. IDH-wildtype, *TERT* promoter wildtype, 1p/19q wildtype), *TP* triple positive (i.e. IDH-mutation, *TERT* promoter mutation and 1p/19q co-deletion)

*P < 0.05, **significant after adjustment for multiple comparisons

### Pathway analysis

To potentially gain further insight into the biological basis of subtype associations, we performed a gene-set enrichment analysis (GSEA) analysing gene expression data from TCGA (Supplementary Table 7 [Online Resource 4]). While we did not identify any significantly altered gene sets (at FDR *q* value < 0.1), the most significantly expressed genes in subgroups was upregulation of PI3K signalling shown in 1p/19q co-deleted tumours (Supplementary Table 7 [Online Resource 4]).

## Discussion

Our findings provide further support for subtype-specific associations for glioma risk loci. Specifically, we confirm the strong relationship between the 8q24.21 (rs55705857) risk variant and Triple-positive glioma. Moreover, we substantiate the proposed specific associations between 5p15.33 (rs10069690) and 20q13.33 (rs2297440) variants with *TERT* promoter mutations, 9p21.3 (rs634537) with *TERT*-only glioma, as well as 17p13.1 (rs78378222) with *TERT*-IDH glioma. Other loci such as 1q32.1 (rs4252707) and 10q25.2 (rs11196067) appear to have more generic effects.

**Fig. 3:**
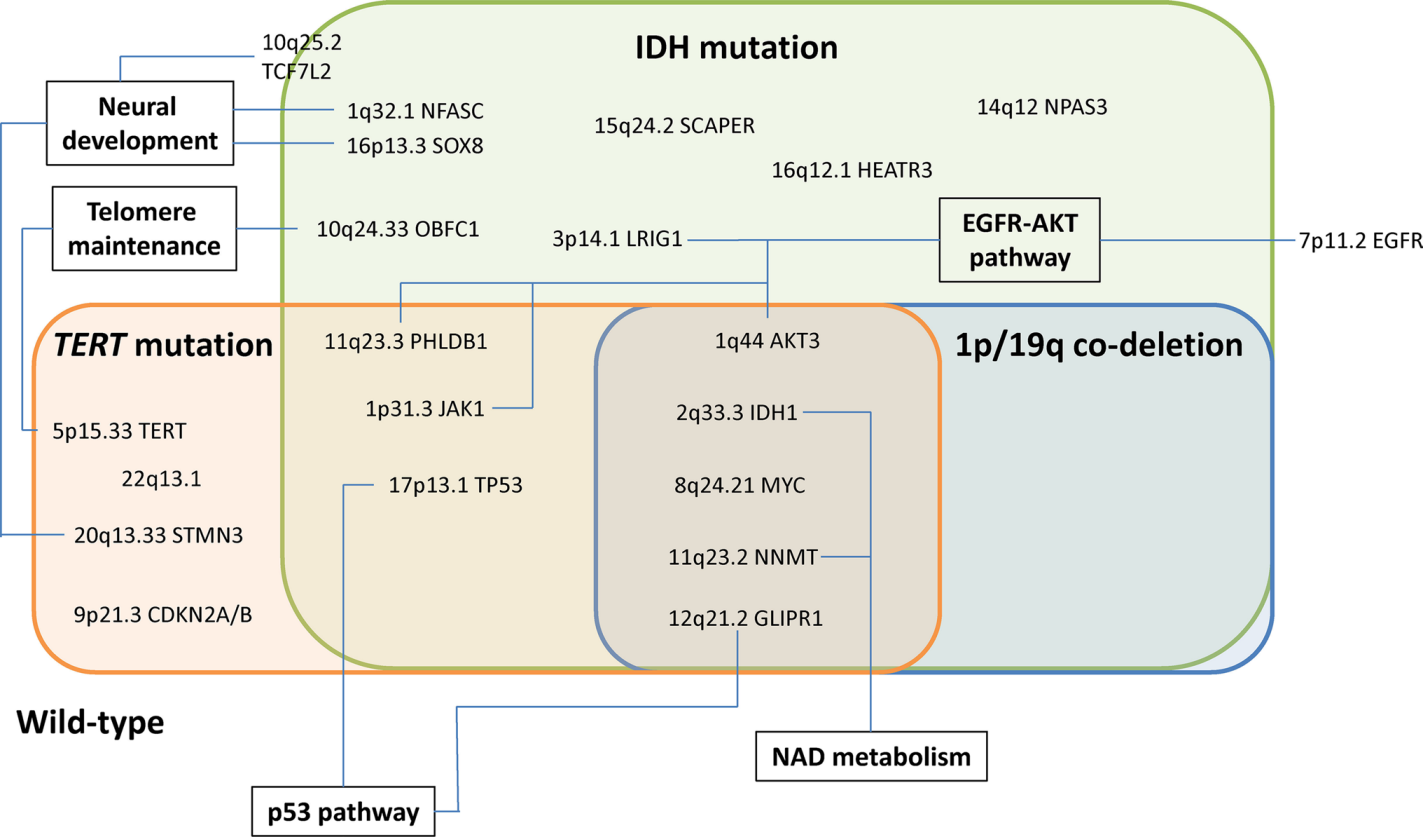
Summary of the relationship between glioma risk with molecular subgroup and associated biological pathways. The extent of the evidence supporting each candidate gene (ranging from an established role in glioma to largely speculative) is summarised in Table 3

Although preliminary, and in part speculative, our analysis delineates potential candidate disease mechanisms across the 25 glioma risk loci (Table 3; Fig. 3). First, maintenance of telomeres is central to cell immortalization [57], and is generally considered to require mutually exclusive mutations in either the *TERT* promoter or *ATRX*. The risk alleles at 5p15.33 (*TERT*) and 10q24.33 (*OBFC1*) are associated with increased leukocyte telomere length, thereby supporting a relationship between SNP genotype and biology [56, 57, 66]. While dysregulation of the telomere gene *RTEL1* has traditionally been assumed to represent the functional basis of the 20q13.33 locus, the glioma risk SNP does not map to the locus associated with telomere length [7, 35]. Intriguingly, our analysis instead implicates *STMN3* at 20q13.33, whose over-expression promotes growth in GBM cells [68], suggesting an alternative mechanism by which the risk SNP influences glioma development. With respect to the 5p15.33 (*TERT*) and 10q24.33 (*OBFC1*) loci, it is unclear whether the effect on glioma risk is solely due to telomeres or is pleiotropic and involves multiple factors. For example, rs10069690 at 5p15.33 is strongly associated with *TERT*-only glioma, yet the *TERT* promoter mutation increases telomerase activity without necessarily affecting telomere length [6]. An intriguing hypothesis to test would, therefore, be to examine the impact of allele-specific effects of rs10069690 on telomere length in the context of gliomas carrying the *TERT* promoter mutation.

Second, the *EGFR*-*AKT* pathway involves *EGFR* at 7p11.2, *LRIG1* at 3p14.1, *PHLDB1* at 11q23.3 and *AKT3* at 1q44. We showed a significant interaction between the risk SNP rs11979158 at 7p11.2 and *EGFR*, consistent with a cis-regulatory effect on gene expression. Although the mechanistic basis of the 7p11.2 locus has long been suspected to involve *EGFR* and is highly associated with classical GBM, emerging evidence suggests that additional components of the *EGFR*-*AKT* signalling pathway are implicated by non-GBM SNPs. At the IDH-only associated locus 3p14.1, *LRIG1* is highly expressed in the brain and negatively regulates the epidermal growth factor receptor (EGFR) signalling pathway [18]. Reduced *LRIG1* expression is linked to tumour aggressiveness, temozolomide resistance and radio-resistance [60, 65]. Downstream components of *EGFR*-*AKT* signalling are implicated at 11q23.3 via *PHLDB1*, as well as 1p31.3 via *JAK1* and 1q44 via *AKT3*. The risk allele of rs12803321 is associated with increased expression of *PHLDB1*, an insulin-responsive protein that enhances Akt activation [70]. *AKT3* at 1q44 is highly expressed in the brain and appears to respond to EGF in a PI3K dependent manner [38], with GBM cells containing amplified AKT3 having enhanced DNA repair and resistance to radiation and temozolomide [54]. The risk allele of rs12752552 at 1p31.3 is associated with increased *JAK1* expression in brain tissue. Since *JAK1* can be activated by EGF phosphorylation, it may be involved in astrocyte formation [3, 39, 50]. The 3p14.1 and 11q23.3 loci are strongly associated with *EGFR* amplification negative gliomas, with a consistent albeit non-significant trend at 1p31.3 and 1q44, consistent with elevated upstream *EGFR* activation masking their functional effects.

Third, the *NAD* pathway involves *IDH1* at 2q33.3 and *NNMT* at 11q23.2. At 2q33.3 we detected a significant Hi-C interaction between the glioma risk SNP rs7572263 and *IDH1*/*IDH1*-*AS1.* Overexpression of *IDH1* mutant proteins has been reported to sensitize glioma cells to radiation [29], providing an interesting mechanism to test the allele-specific effects of this SNP. IDH mutation causes de-regulation of NAD signalling [64]. Interestingly, therefore, at 11q23.2 which is strongly associated with IDH mutated gliomas, the most convincing molecular mechanism is via *NNMT*, which encodes nicotinamide *N*-methyltransferase and is highly expressed in GBM relative to normal brain, causing methionine depletion-mediated DNA hypomethylation and accelerated tumour growth [23, 55].

Fourth, genes with established roles in neural development may be involved. While the risk SNP rs4252707 at 1q32.1 is within the intron of *MDM4*, the strongest evidence for a mechanistic effect was with *NFASC*. Neurofascin is involved in synapse formation during neural development [1] and, therefore, represents an attractive functional candidate for the association with glioma. Additionally at 16p13.3 and 20q13.33, implicated genes *SOX8* and *STMN3* are strongly expressed in the brain and thought to play a role in neural development [47, 68]. At 10q25.2, implicated gene *TCF7L2* modifies beta-catenin signalling and controls oligodendrocyte differentiation [69]. Intriguingly, 10q25.2 has previously been reported to be a risk locus for colorectal cancer [58], a tumour driven by wnt signalling, however, the risk SNP is not correlated with rs11196067 raising the possibility of tissue-specific regulation across the wider region.

Finally, the p53 pathway is involved at 17p13.1, where the risk SNP rs78378222 affects *TP53* 3′UTR poly-adenylation processing. In addition, the p53 target GLIPR1 [43] is implicated at 12q21.2. Moreover, 12q21.2 is most strongly associated with Triple-positive glioma, which does not feature *TP53* mutation, consistent with wild-type p53 protein being required for the SNP to exert a functional effect.

As with many cancers, the exact point at which the risk SNPs exert their functional impact on glioma oncogenesis still remains to be elucidated, and we did not demonstrate a relationship between increased risk allele number and age at diagnosis. Surprisingly we found a significant association between increasing risk allele number and improved outcome. This result was consistent across the prognostic molecular groups, consistent with our observations not being due to an over-representation of the more favourable prognostic groups among patients with a higher burden of risk alleles. In addition, the distribution of risk allele numbers did not differ across the four groups (*P* = 0.3, ANOVA test). Examining the impact of an individual SNP’s impact on survival did not reveal any loci strongly associated with outcome. Collectively our findings suggest that, independent of other prognostic factors, the greater the number of risk alleles carried, the better the outcome.

In conclusion, we performed the most comprehensive association study between molecular subgroup and the 25 recently identified glioma risk loci to date. While confirming previous observations, we show that the majority of risk loci are associated with IDH mutation. Through the integration of Hi-C and eQTL data, we have additionally sought to define candidate target genes underlying the associations. Collectively our observations highlight pathways critical to glioma susceptibility, notably neural development and NAD metabolism, as well as EGFR-AKT signalling. Intriguingly, we show here that the number of risk alleles is consistently associated with better outcome. Functional investigation in tumour and neural progenitor-based systems will be required to more fully elucidate these molecular mechanisms. Notably, IDH mutant tumours have been shown to reshape 3D chromatin organisation and may reveal new regulatory interactions [14].

Our current analysis is based on defining glioma subgroups using only three primary markers. Given the extent of the missing heritability for glioma further expansion of GWAS by international consortia [35] is likely to result in the identification of additional risk variants. Additional molecular sub-grouping glioma resulting from ongoing large-scale tumour sequencing projects is likely to provide for further insights into glial oncogenesis and ultimately may suggest targets for novel therapeutic strategies.

## Electronic supplementary material

Below is the link to the electronic supplementary material.

Supplementary material 1 (PDF 6784 kb)

Supplementary material 2 (XLSX 120 kb)

Supplementary material 3 (XLSX 16 kb)

Supplementary material 4 (XLSX 85 kb)

## References

[1] Ango F, di Cristo G, Higashiyama H, Bennett V, Wu P, Huang ZJ (2004). Ankyrin-based subcellular gradient of neurofascin, an immunoglobulin family protein, directs GABAergic innervation at purkinje axon initial segment. Cell.

[2] Bondy ML, Scheurer ME, Malmer B, Barnholtz-Sloan JS, Davis FG, Il’yasova D, Kruchko C, McCarthy BJ, Rajaraman P, Schwartzbaum JA (2008). Brain tumor epidemiology: consensus from the Brain Tumor Epidemiology Consortium. Cancer.

[3] Bonni A, Sun Y, Nadal-Vicens M, Bhatt A, Frank DA, Rozovsky I, Stahl N, Yancopoulos GD, Greenberg ME (1997). Regulation of gliogenesis in the central nervous system by the JAK-STAT signaling pathway. Science.

[4] Brodbeck D, Cron P, Hemmings BA (1999). A human protein kinase Bgamma with regulatory phosphorylation sites in the activation loop and in the C-terminal hydrophobic domain. J Biol Chem.

[5] Brat DJ, Verhaak RG, Aldape KD, Yung WK, Salama SR, Cooper LA, Rheinbay E, Miller CR, Vitucci M, Cancer Genome Atlas Research Network (2015). Comprehensive, integrative genomic analysis of diffuse lower-grade gliomas. N Engl J Med.

[6] Ceccarelli M, Barthel FP, Malta TM, Sabedot TS, Salama SR, Murray BA, Morozova O, Newton Y, Radenbaugh A, Pagnotta SM (2016). Molecular profiling reveals biologically discrete subsets and pathways of progression in diffuse glioma. Cell.

[7] Codd V, Nelson CP, Albrecht E, Mangino M, Deelen J, Buxton JL, Hottenga JJ, Fischer K, Esko T, Surakka I (2013). Identification of seven loci affecting mean telomere length and their association with disease. Nat Genet.

[8] Crane E, Bian Q, McCord RP, Lajoie BR, Wheeler BS, Ralston EJ, Uzawa S, Dekker J, Meyer BJ (2015). Condensin-driven remodelling of X chromosome topology during dosage compensation. Nature.

[9] Crocetti E, Trama A, Stiller C, Caldarella A, Soffietti R, Jaal J, Weber DC, Ricardi U, Slowinski J, Brandes A (2012). Epidemiology of glial and non-glial brain tumours in Europe. Eur J Cancer.

[10] Di Stefano AL, Enciso-Mora V, Marie Y, Desestret V, Labussiere M, Boisselier B, Mokhtari K, Idbaih A, Hoang-Xuan K, Delattre JY (2013). Association between glioma susceptibility loci and tumour pathology defines specific molecular etiologies. Neuro Oncol.

[11] Dixon JR, Jung I, Selvaraj S, Shen Y, Antosiewicz-Bourget JE, Lee AY, Ye Z, Kim A, Rajagopal N, Xie W (2015). Chromatin architecture reorganization during stem cell differentiation. Nature.

[12] Eckel-Passow JE, Lachance DH, Molinaro AM, Walsh KM, Decker PA, Sicotte H, Pekmezci M, Rice T, Kosel ML, Smirnov IV (2015). Glioma groups based on 1p/19q, IDH, and TERT promoter mutations in tumors. N Engl J Med.

[13] Enciso-Mora V, Hosking FJ, Kinnersley B, Wang Y, Shete S, Zelenika D, Broderick P, Idbaih A, Delattre JY, Hoang-Xuan K (2013). Deciphering the 8q24.21 association for glioma. Hum Mol Genet.

[14] Flavahan WA, Drier Y, Liau BB, Gillespie SM, Venteicher AS, Stemmer-Rachamimov AO, Suva ML, Bernstein BE (2016). Insulator dysfunction and oncogene activation in IDH mutant gliomas. Nature.

[15] Gao J, Aksoy BA, Dogrusoz U, Dresdner G, Gross B, Sumer SO, Sun Y, Jacobsen A, Sinha R, Larsson E et al (2013) Integrative analysis of complex cancer genomics and clinical profiles using the cBioPortal. Sci Signal 6:pl1. http://stke.sciencemag.org/content/6/269/pl1.full 10.1126/scisignal.2004088 PMC4160307 23550210

[16] Gonzalez-Aguilar A, Idbaih A, Boisselier B, Habbita N, Rossetto M, Laurenge A, Bruno A, Jouvet A, Polivka M, Adam C (2012). Recurrent mutations of MYD88 and TBL1XR1 in primary central nervous system lymphomas. Clin Cancer Res Off J Am Assoc Cancer Res.

[17] Group CS (2003). Vascular factors and risk of dementia: design of the Three-City Study and baseline characteristics of the study population. Neuroepidemiology.

[18] Gur G, Rubin C, Katz M, Amit I, Citri A, Nilsson J, Amariglio N, Henriksson R, Rechavi G, Hedman H (2004). LRIG1 restricts growth factor signaling by enhancing receptor ubiquitylation and degradation. EMBO J.

[19] Hercberg S, Galan P, Preziosi P, Bertrais S, Mennen L, Malvy D, Roussel AM, Favier A, Briancon S (2004). The SU. VI. MAX Study: a randomized, placebo-controlled trial of the health effects of antioxidant vitamins and minerals. Arch Intern Med.

[20] Higgins JP, Thompson SG, Deeks JJ, Altman DG (2003). Measuring inconsistency in meta-analyses. BMJ.

[21] Idbaih A, Marie Y, Lucchesi C, Pierron G, Manie E, Raynal V, Mosseri V, Hoang-Xuan K, Kujas M, Brito I (2008). BAC array CGH distinguishes mutually exclusive alterations that define clinicogenetic subtypes of gliomas. Int J Cancer J Int Cancer.

[22] Jenkins RB, Xiao Y, Sicotte H, Decker PA, Kollmeyer TM, Hansen HM, Kosel ML, Zheng S, Walsh KM, Rice T (2012). A low-frequency variant at 8q24.21 is strongly associated with risk of oligodendroglial tumors and astrocytomas with IDH1 or IDH2 mutation. Nat Genet.

[23] Jung J, Kim LJY, Wang X, Wu Q, Sanvoranart T, Hubert CG, Prager BC, Wallace LC, Jin X, Mack SC et al (2017) Nicotinamide metabolism regulates glioblastoma stem cell maintenance. JCI Insight 2. 10.1172/jci.insight.90019 10.1172/jci.insight.90019 PMC5436539 28515364

[24] Killedar A, Stutz MD, Sobinoff AP, Tomlinson CG, Bryan TM, Beesley J, Chenevix-Trench G, Reddel RR, Pickett HA (2015). A common cancer risk-associated allele in the hTERT locus encodes a dominant negative inhibitor of telomerase. PLoS Genet.

[25] Kinnersley B, Labussiere M, Holroyd A, Di Stefano AL, Broderick P, Vijayakrishnan J, Mokhtari K, Delattre JY, Gousias K, Schramm J (2015). Genome-wide association study identifies multiple susceptibility loci for glioma. Nat Commun.

[26] Labussiere M, Di Stefano AL, Gleize V, Boisselier B, Giry M, Mangesius S, Bruno A, Paterra R, Marie Y, Rahimian A (2014). TERT promoter mutations in gliomas, genetic associations and clinico-pathological correlations. Br J Cancer.

[27] Labussiere M, Idbaih A, Wang XW, Marie Y, Boisselier B, Falet C, Paris S, Laffaire J, Carpentier C, Criniere E (2010). All the 1p19q codeleted gliomas are mutated on IDH1 or IDH2. Neurology.

[28] Lee JH, Huynh M, Silhavy JL, Kim S, Dixon-Salazar T, Heiberg A, Scott E, Bafna V, Hill KJ, Collazo A (2012). De novo somatic mutations in components of the PI3 K-AKT3-mTOR pathway cause hemimegalencephaly. Nat Genet.

[29] Li S, Chou AP, Chen W, Chen R, Deng Y, Phillips HS, Selfridge J, Zurayk M, Lou JJ, Everson RG (2013). Overexpression of isocitrate dehydrogenase mutant proteins renders glioma cells more sensitive to radiation. Neuro Oncol.

[30] Liu JZ, Tozzi F, Waterworth DM, Pillai SG, Muglia P, Middleton L, Berrettini W, Knouff CW, Yuan X, Waeber G (2010). Meta-analysis and imputation refines the association of 15q25 with smoking quantity. Nat Genet.

[31] Lonsdale J, Thomas J, Salvatore M, Phillips R, Lo E, Shad S, Hasz R, Walters G, Garcia F, Young N (2013). The genotype-tissue expression (GTEx) project. Nat Genet.

[32] Louis DN, Perry A, Reifenberger G, von Deimling A, Figarella-Branger D, Cavenee WK, Ohgaki H, Wiestler OD, Kleihues P, Ellison DW (2016). The 2016 World Health Organization classification of tumors of the central nervous system: a summary. Acta Neuropathol.

[33] Marchini J, Howie B, Myers S, McVean G, Donnelly P (2007). A new multipoint method for genome-wide association studies by imputation of genotypes. Nat Genet.

[34] Martin JS, Xu Z, Reiner AP, Mohlke KL, Sullivan P, Ren B, Hu M, Li Y (2017). HUGIn: Hi-C unifying genomic interrogator. Bioinformatics.

[35] Melin BS, Barnholtz-Sloan JS, Wrensch MR, Johansen C, Il’yasova D, Kinnersley B, Ostrom QT, Labreche K, Chen Y, Armstrong G (2017). Genome-wide association study of glioma subtypes identifies specific differences in genetic susceptibility to glioblastoma and non-glioblastoma tumors. Nat Genet.

[36] Mootha VK, Lindgren CM, Eriksson KF, Subramanian A, Sihag S, Lehar J, Puigserver P, Carlsson E, Ridderstrale M, Laurila E (2003). PGC-1alpha-responsive genes involved in oxidative phosphorylation are coordinately downregulated in human diabetes. Nat Genet.

[37] Moreira F, Kiehl TR, So K, Ajeawung NF, Honculada C, Gould P, Pieper RO, Kamnasaran D (2011). NPAS3 demonstrates features of a tumor suppressive role in driving the progression of Astrocytomas. Am J Pathol.

[38] Okano J, Gaslightwala I, Birnbaum MJ, Rustgi AK, Nakagawa H (2000). Akt/protein kinase B isoforms are differentially regulated by epidermal growth factor stimulation. J Biol Chem.

[39] Park OK, Schaefer TS, Nathans D (1996). In vitro activation of Stat3 by epidermal growth factor receptor kinase. Proc Natl Acad Sci USA.

[40] Patterson N, Price AL, Reich D (2006). Population structure and eigenanalysis. PLoS Genet.

[41] Power C, Elliott J (2006). Cohort profile: 1958 British birth cohort (National Child Development Study). Int J Epidemiol.

[42] Rao SS, Huntley MH, Durand NC, Stamenova EK, Bochkov ID, Robinson JT, Sanborn AL, Machol I, Omer AD, Lander ES (2014). A 3D map of the human genome at kilobase resolution reveals principles of chromatin looping. Cell.

[43] Ren C, Ren CH, Li L, Goltsov AA, Thompson TC (2006). Identification and characterization of RTVP1/GLIPR1-like genes, a novel p53 target gene cluster. Genomics.

[44] Sanson M, Hosking FJ, Shete S, Zelenika D, Dobbins SE, Ma Y, Enciso-Mora V, Idbaih A, Delattre JY, Hoang-Xuan K (2011). Chromosome 7p11.2 (EGFR) variation influences glioma risk. Hum Mol Genet.

[45] Sanson M, Marie Y, Paris S, Idbaih A, Laffaire J, Ducray F, El Hallani S, Boisselier B, Mokhtari K, Hoang-Xuan K (2009). Isocitrate dehydrogenase 1 codon 132 mutation is an important prognostic biomarker in gliomas. J Clin Oncol Off J Am Soc Clin Oncol.

[46] Sanz-Moreno V, Gaggioli C, Yeo M, Albrengues J, Wallberg F, Viros A, Hooper S, Mitter R, Feral CC, Cook M (2011). ROCK and JAK1 signaling cooperate to control actomyosin contractility in tumor cells and stroma. Cancer Cell.

[47] Schepers GE, Bullejos M, Hosking BM, Koopman P (2000). Cloning and characterisation of the Sry-related transcription factor gene Sox8. Nucleic Acids Res.

[48] Schmitt AD, Hu M, Jung I, Xu Z, Qiu Y, Tan CL, Li Y, Lin S, Lin Y, Barr CL (2016). A compendium of chromatin contact maps reveals spatially active regions in the human genome. Cell Rep.

[49] Shete S, Hosking FJ, Robertson LB, Dobbins SE, Sanson M, Malmer B, Simon M, Marie Y, Boisselier B, Delattre JY (2009). Genome-wide association study identifies five susceptibility loci for glioma. Nat Genet.

[50] Shuai K, Ziemiecki A, Wilks AF, Harpur AG, Sadowski HB, Gilman MZ, Darnell JE (1993). Polypeptide signalling to the nucleus through tyrosine phosphorylation of Jak and Stat proteins. Nature.

[51] Stacey SN, Sulem P, Jonasdottir A, Masson G, Gudmundsson J, Gudbjartsson DF, Magnusson OT, Gudjonsson SA, Sigurgeirsson B, Thorisdottir K (2011). A germline variant in the TP53 polyadenylation signal confers cancer susceptibility. Nat Genet.

[52] Subramanian A, Tamayo P, Mootha VK, Mukherjee S, Ebert BL, Gillette MA, Paulovich A, Pomeroy SL, Golub TR, Lander ES (2005). Gene set enrichment analysis: a knowledge-based approach for interpreting genome-wide expression profiles. Proc Natl Acad Sci USA.

[53] Sud A, Kinnersley B, Houlston RS (2017). Genome-wide association studies of cancer: current insights and future perspectives. Nat Rev Cancer.

[54] Turner KM, Sun Y, Ji P, Granberg KJ, Bernard B, Hu L, Cogdell DE, Zhou X, Yli-Harja O, Nykter M (2015). Genomically amplified Akt3 activates DNA repair pathway and promotes glioma progression. Proc Natl Acad Sci USA.

[55] Ulanovskaya OA, Zuhl AM, Cravatt BF (2013). NNMT promotes epigenetic remodeling in cancer by creating a metabolic methylation sink. Nat Chem Biol.

[56] Walsh KM, Codd V, Rice T, Nelson CP, Smirnov IV, McCoy LS, Hansen HM, Elhauge E, Ojha J, Francis SS (2015). Longer genotypically-estimated leukocyte telomere length is associated with increased adult glioma risk. Oncotarget.

[57] Walsh KM, Wiencke JK, Lachance DH, Wiemels JL, Molinaro AM, Eckel-Passow JE, Jenkins RB, Wrensch MR (2015). Telomere maintenance and the etiology of adult glioma. Neuro Oncol.

[58] Wang H, Burnett T, Kono S, Haiman CA, Iwasaki M, Wilkens LR, Loo LW, Van Den Berg D, Kolonel LN, Henderson BE (2014). Trans-ethnic genome-wide association study of colorectal cancer identifies a new susceptibility locus in VTI1A. Nat Commun.

[59] Wang K, Li M, Hadley D, Liu R, Glessner J, Grant SF, Hakonarson H, Bucan M (2007). PennCNV: an integrated hidden Markov model designed for high-resolution copy number variation detection in whole-genome SNP genotyping data. Genome Res.

[60] Wei J, Qi X, Zhan Q, Zhou D, Yan Q, Wang Y, Mo L, Wan Y, Xie D, Xie J (2015). miR-20a mediates temozolomide-resistance in glioblastoma cells via negatively regulating LRIG1 expression. Biomed Pharmacother.

[61] Westra HJ, Peters MJ, Esko T, Yaghootkar H, Schurmann C, Kettunen J, Christiansen MW, Fairfax BP, Schramm K, Powell JE (2013). Systematic identification of trans eQTLs as putative drivers of known disease associations. Nat Genet.

[62] Wickham H (2009). ggplot2: elegant graphics for data analysis.

[63] Wrensch M, Jenkins RB, Chang JS, Yeh RF, Xiao Y, Decker PA, Ballman KV, Berger M, Buckner JC, Chang S (2009). Variants in the CDKN2B and RTEL1 regions are associated with high-grade glioma susceptibility. Nat Genet.

[64] Yan H, Parsons DW, Jin G, McLendon R, Rasheed BA, Yuan W, Kos I, Batinic-Haberle I, Jones S, Riggins GJ (2009). IDH1 and IDH2 mutations in gliomas. N Engl J Med.

[65] Yang JA, Liu BH, Shao LM, Guo ZT, Yang Q, Wu LQ, Ji BW, Zhu XN, Zhang SQ, Li CJ (2015). LRIG1 enhances the radiosensitivity of radioresistant human glioblastoma U251 cells via attenuation of the EGFR/Akt signaling pathway. Int J Clin Exp Pathol.

[66] Zhang C, Doherty JA, Burgess S, Hung RJ, Lindstrom S, Kraft P, Gong J, Amos CI, Sellers TA, Monteiro AN (2015). Genetic determinants of telomere length and risk of common cancers: a Mendelian randomization study. Hum Mol Genet.

[67] Zhang W, Hui KY, Gusev A, Warner N, Ng SM, Ferguson J, Choi M, Burberry A, Abraham C, Mayer L (2013). Extended haplotype association study in Crohn’s disease identifies a novel, Ashkenazi Jewish-specific missense mutation in the NF-kappaB pathway gene, HEATR3. Genes Immun.

[68] Zhang Y, Ni S, Huang B, Wang L, Zhang X, Li X, Wang H, Liu S, Hao A (2015). Overexpression of SCLIP promotes growth and motility in glioblastoma cells. Cancer Biol Ther.

[69] Zhao C, Deng Y, Liu L, Yu K, Zhang L, Wang H, He X, Wang J, Lu C, Wu LN (2016). Dual regulatory switch through interactions of Tcf7l2/Tcf4 with stage-specific partners propels oligodendroglial maturation. Nat Commun.

[70] Zhou QL, Jiang ZY, Mabardy AS, Del Campo CM, Lambright DG, Holik J, Fogarty KE, Straubhaar J, Nicoloro S, Chawla A (2010). A novel pleckstrin homology domain-containing protein enhances insulin-stimulated Akt phosphorylation and GLUT4 translocation in adipocytes. J Biol Chem.

