# Supplementary material for: Diffuse gliomas classified by 1p/19q co-deletion, TERT promoter and IDH mutation status are associated with specific genetic risk loci
Source: Acta Neuropathol. 2018 Feb 19;135(5):743–55. doi: 10.1007/s00401-018-1825-z (PMC5904227; doi:10.1007/s00401-018-1825-z)
Supplement: Supplementary file 1 — Supplementary material 1 (PDF 6784 kb) [file 401_2018_1825_MOESM1_ESM.pdf]

Supplementary Figure 1: Identification of individuals of non-European ancestry in TCGA cases and WTCCC controls. (a) Before excluding non-European ancestry in cases and controls; (b) after. The first two principal components of the analysis are plotted. HapMap CEU individuals are plotted in red, JPT individuals are plotted in pink, CHB are plotted in cyan, YRI are plotted in yellow. Cases are plotted in green and controls are plotted in blue.

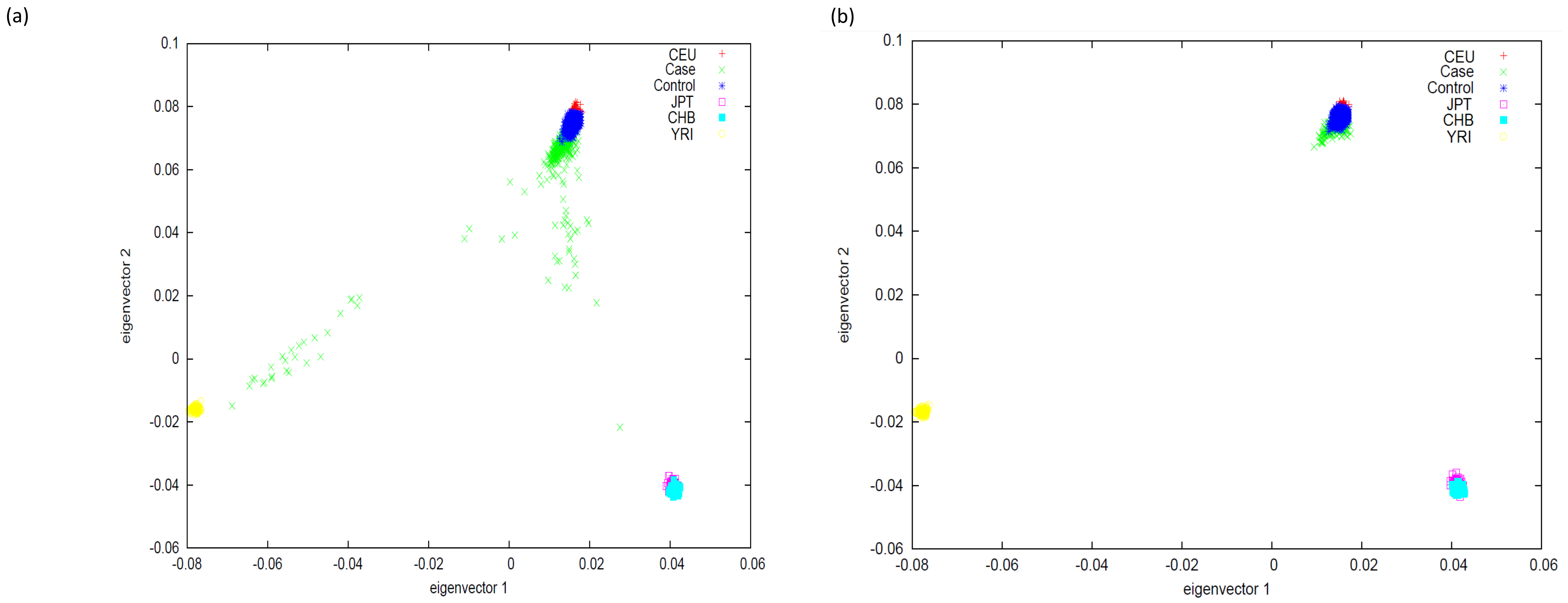

Supplementary Figure 2: Quantile-Quantile (Q-Q) plots of observed and expected χ^2 values of association between SNP genotype and risk of glioma after imputation using TCGA cases and WTCCC controls. (a) Before adjustment; (b) after adjustment for population stratification using the top ten principal components of genetic variation between cases and controls. The red line represents the null hypothesis of no true association.

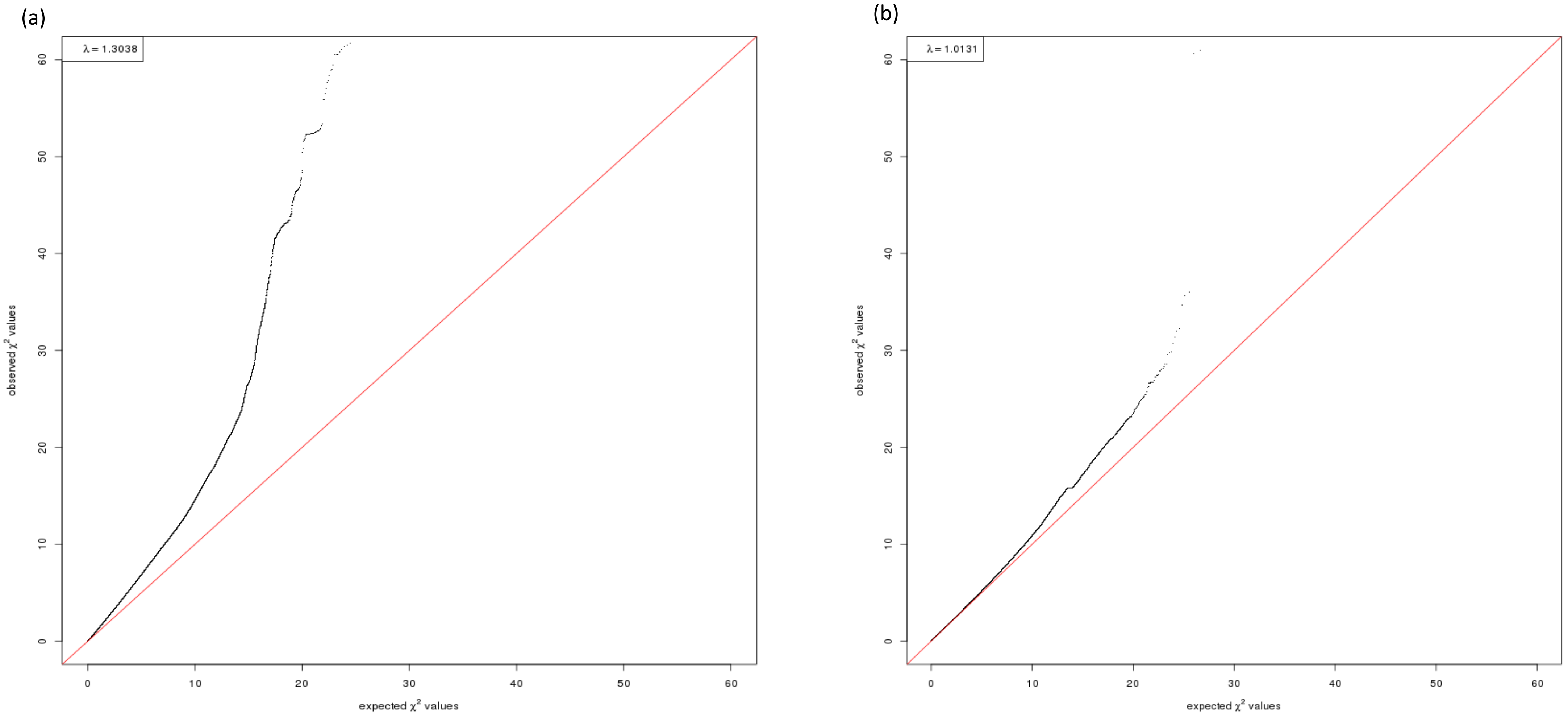

Supplementary Figure 3: Survival by molecular subgroup. (a) All patients cohorts combined – all glioma; (b) French GWAS – all glioma; (c) French sequenced – all glioma; (d) TCGA- all glioma; (e) All dataset combined – GBM; (f) French GWAS – GBM; (g) French sequenced – GBM; (h) TCGA – GBM; (i) All dataset combined – nonGBM; (j) French GWAS – non-GBM; (k) French sequenced – non-GBM; (l) TCGA non-GBM

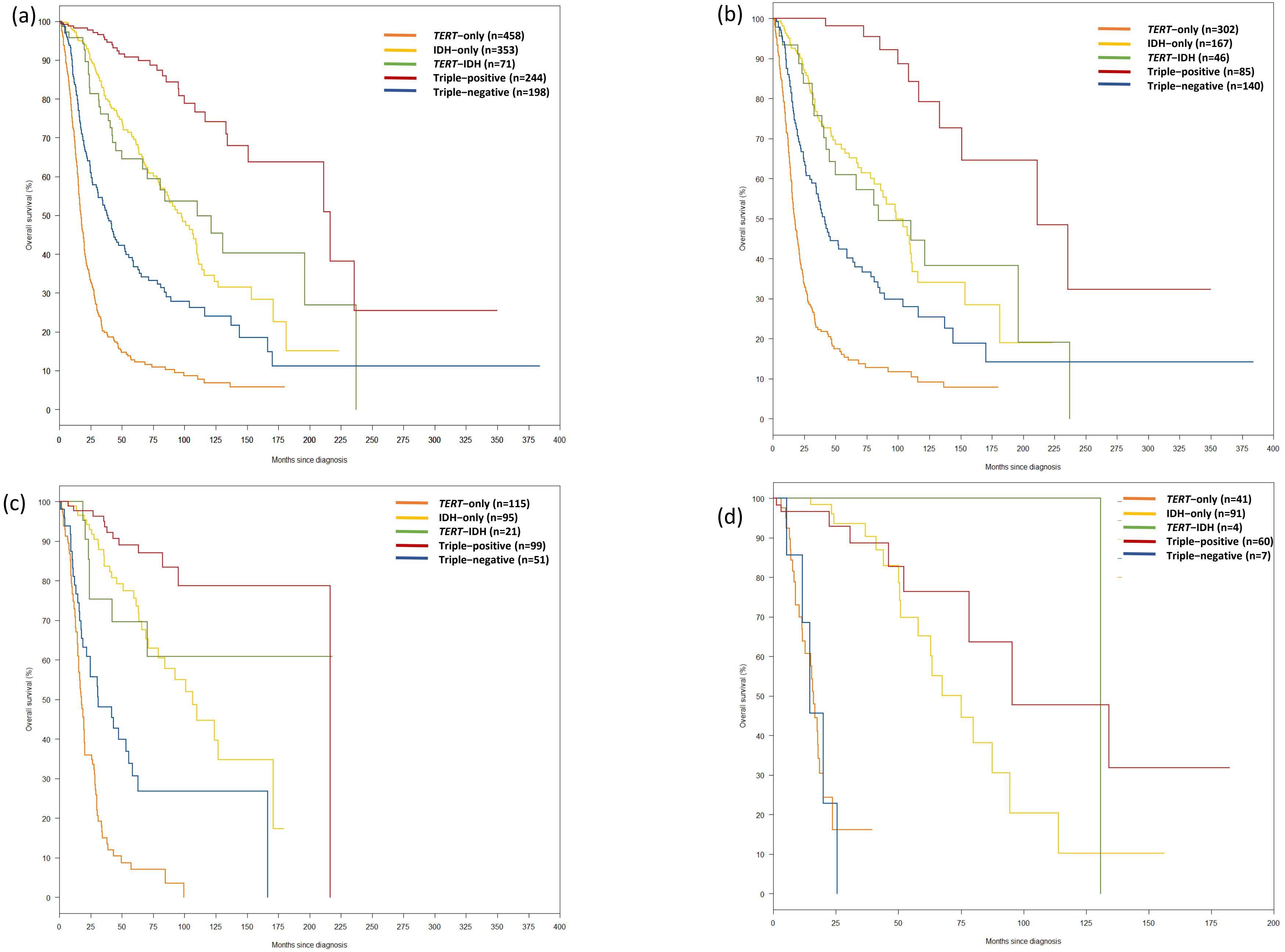

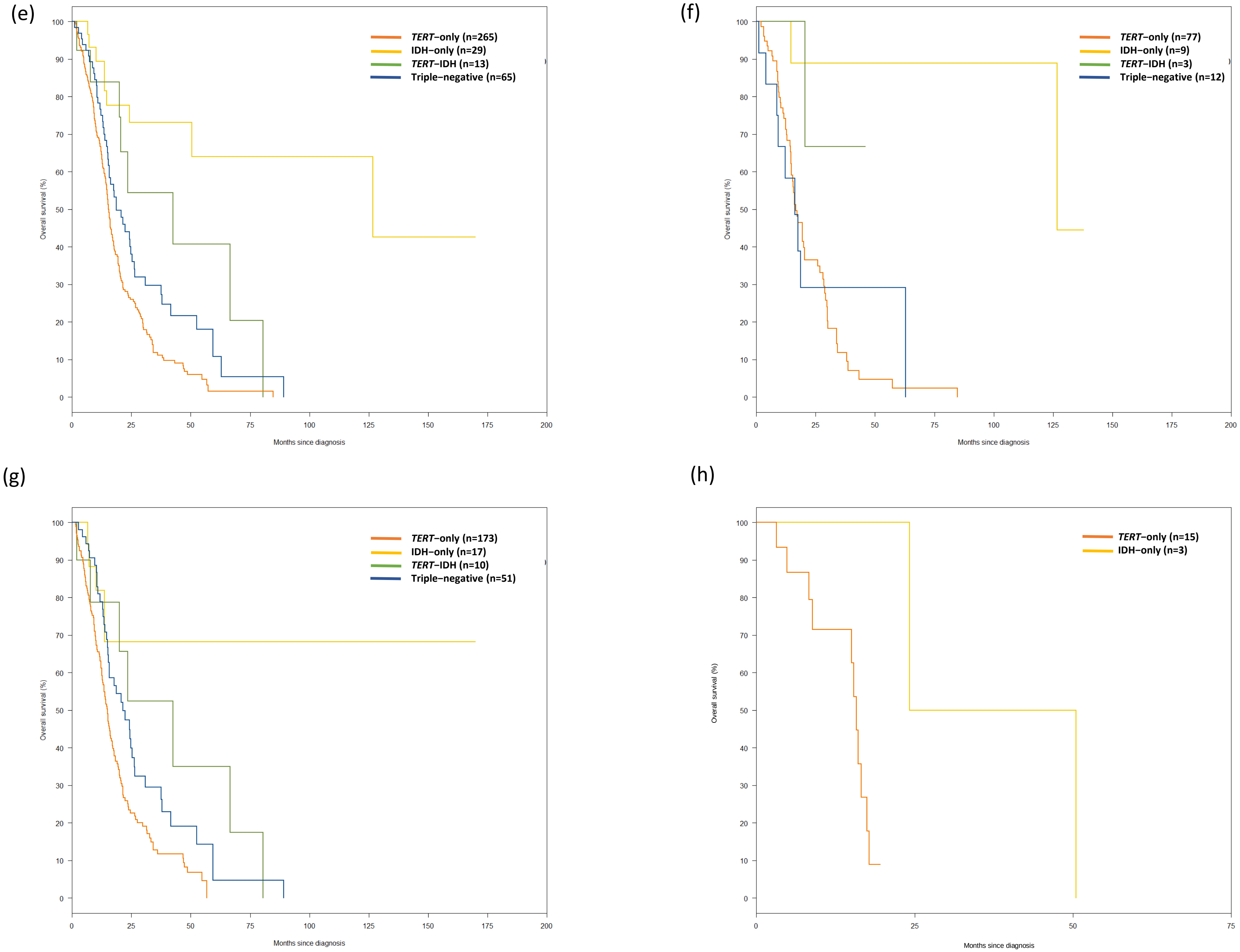
(e)
TERT–only (n=265)
IDH–only (n=29)
TERT–IDH (n=13)
Triple–negative (n=65)
Overall survival (%)
Months since diagnosis
(f)
TERT–only (n=77)
IDH–only (n=9)
TERT–IDH (n=3)
Triple–negative (n=12)
Overall survival (%)
Months since diagnosis
(g)
TERT–only (n=173)
IDH–only (n=17)
TERT–IDH (n=10)
Triple–negative (n=51)
Overall survival (%)
Months since diagnosis
(h)
TERT–only (n=15)
IDH–only (n=3)
Overall survival (%)
Months since diagnosis

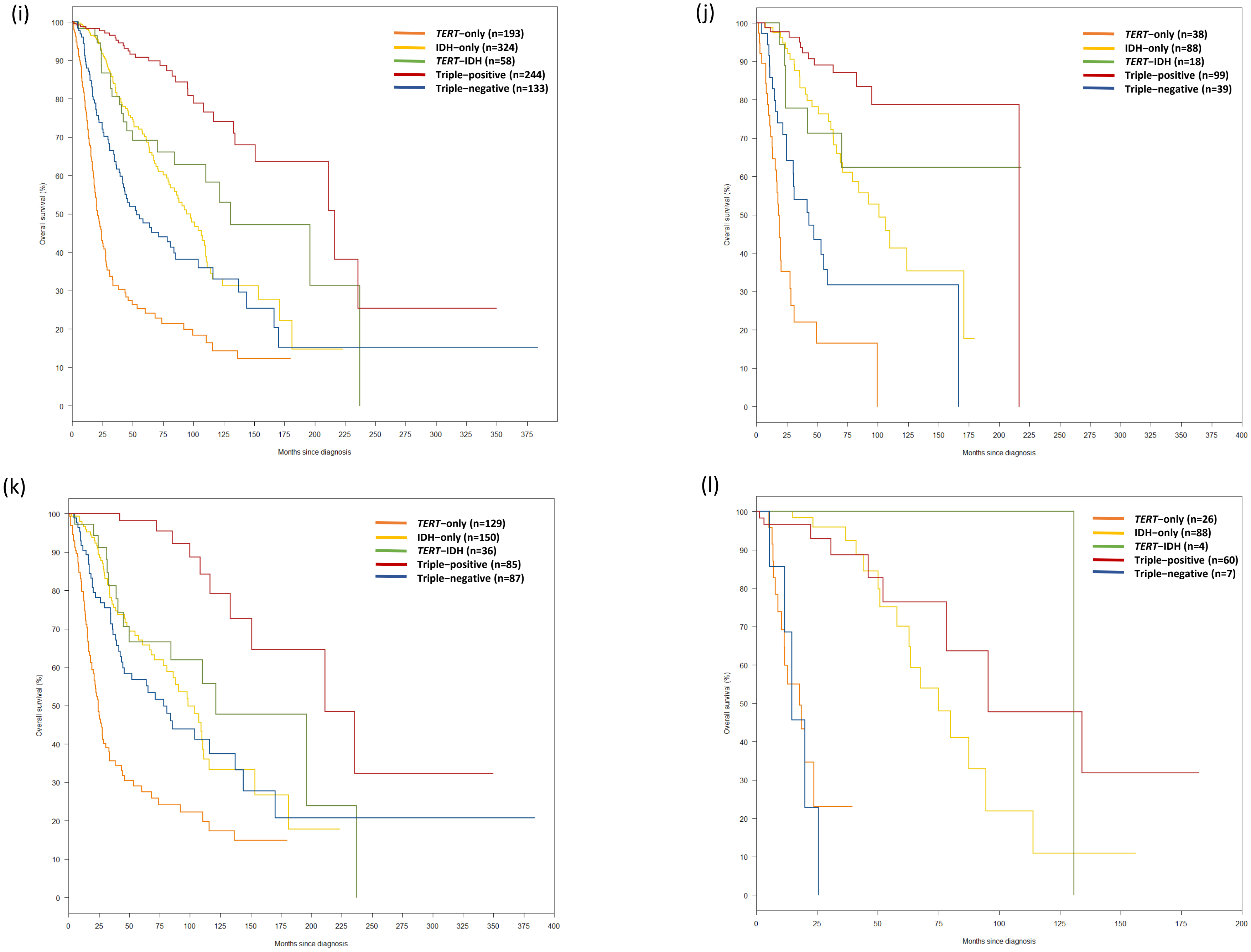

(i)
TERT–only (n=193)
IDH–only (n=324)
TERT–IDH (n=58)
Triple–positive (n=244)
Triple–negative (n=133)
Overall survival (%)
Months since diagnosis
(j)
TERT–only (n=38)
IDH–only (n=88)
TERT–IDH (n=18)
Triple–positive (n=99)
Triple–negative (n=39)
Overall survival (%)
Months since diagnosis
(k)
TERT–only (n=129)
IDH–only (n=150)
TERT–IDH (n=36)
Triple–positive (n=85)
Triple–negative (n=87)
Overall survival (%)
Months since diagnosis
(l)
TERT–only (n=26)
IDH–only (n=88)
TERT–IDH (n=4)
Triple–positive (n=60)
Triple–negative (n=7)
Overall survival (%)
Months since diagnosis

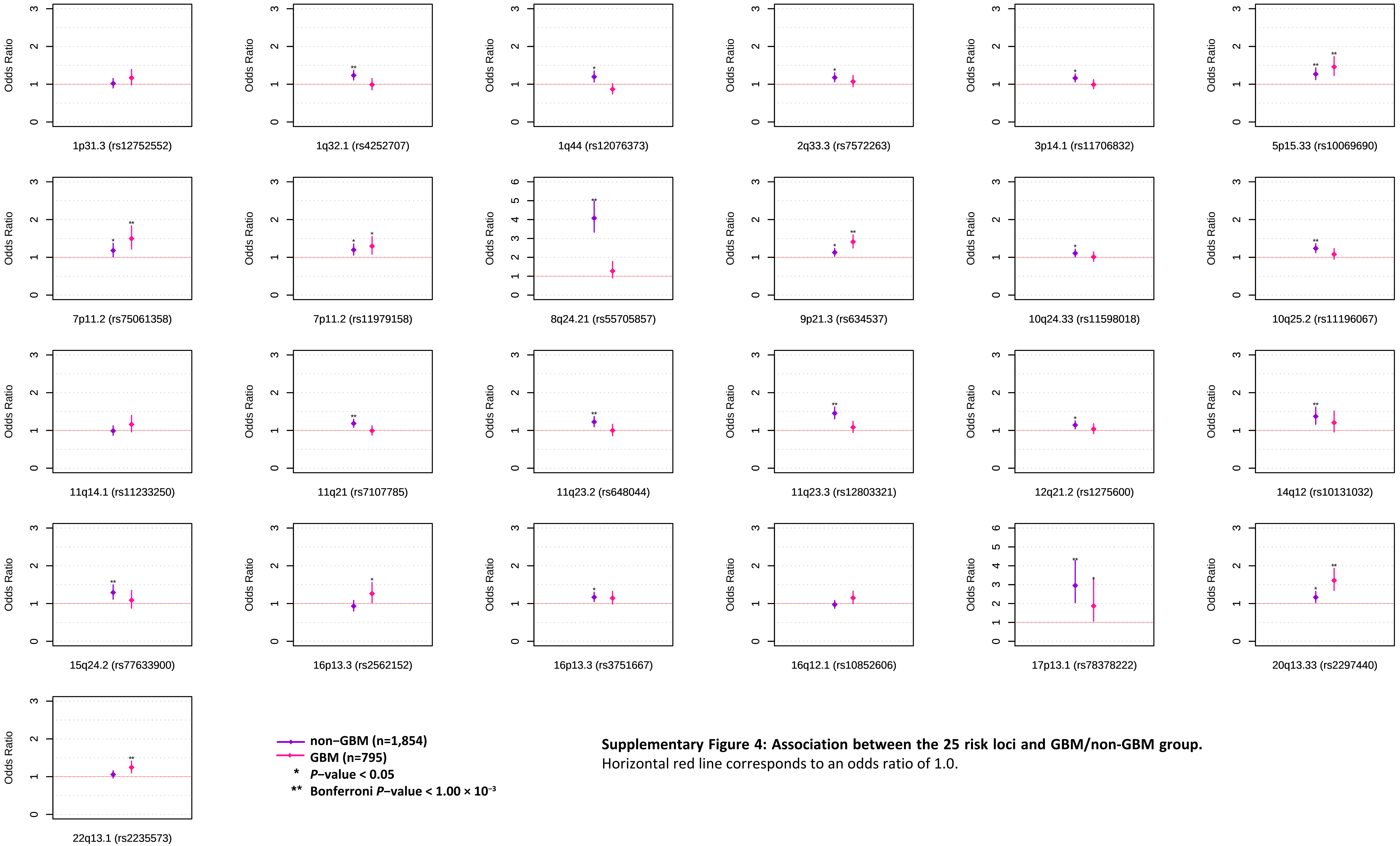

Supplementary Figure 4: Association between the 25 risk loci and GBM/non-GBM group.
Horizontal red line corresponds to an odds ratio of 1.0.

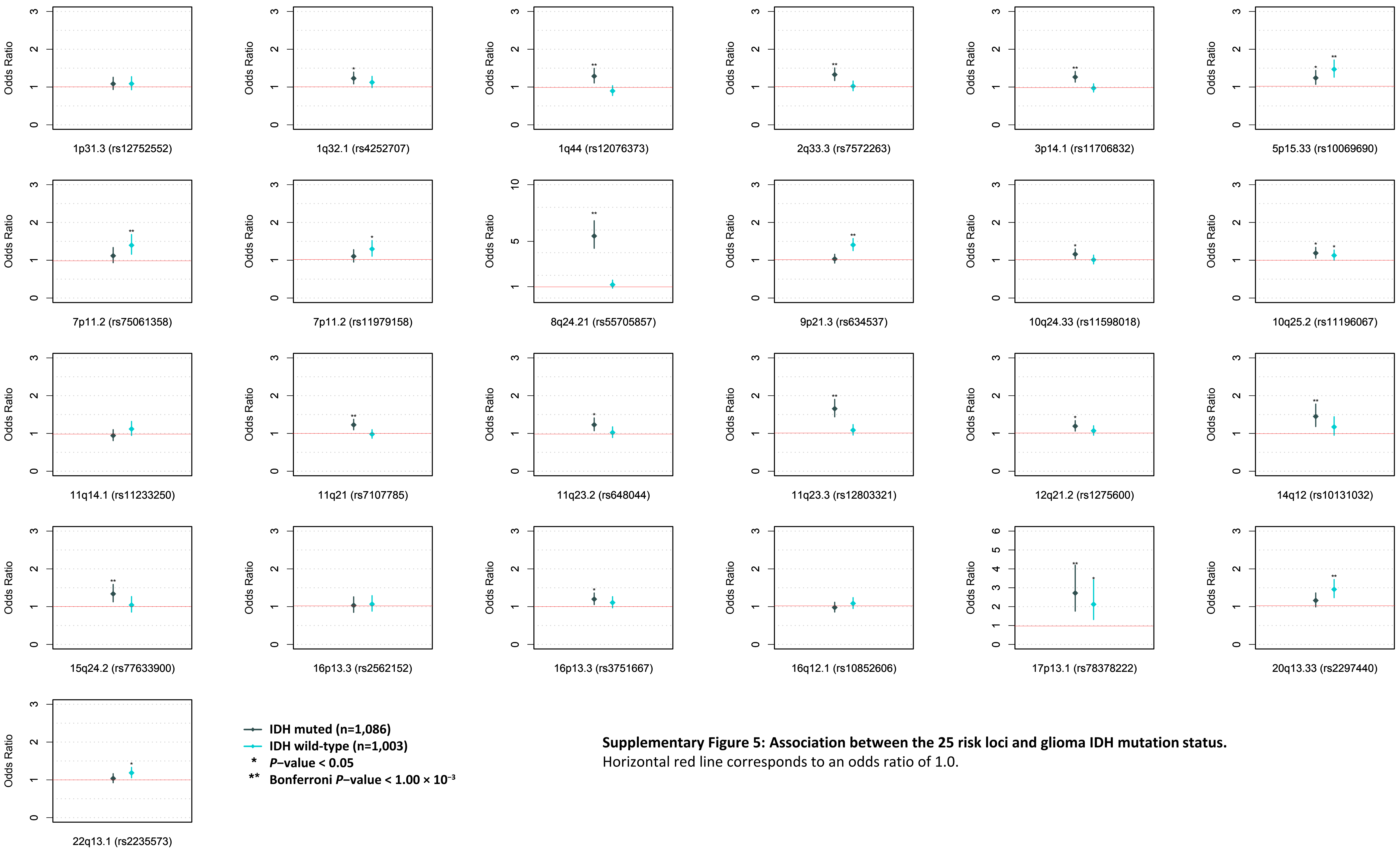

Supplementary Figure 5: Association between the 25 risk loci and glioma IDH mutation status. Horizontal red line corresponds to an odds ratio of 1.0.

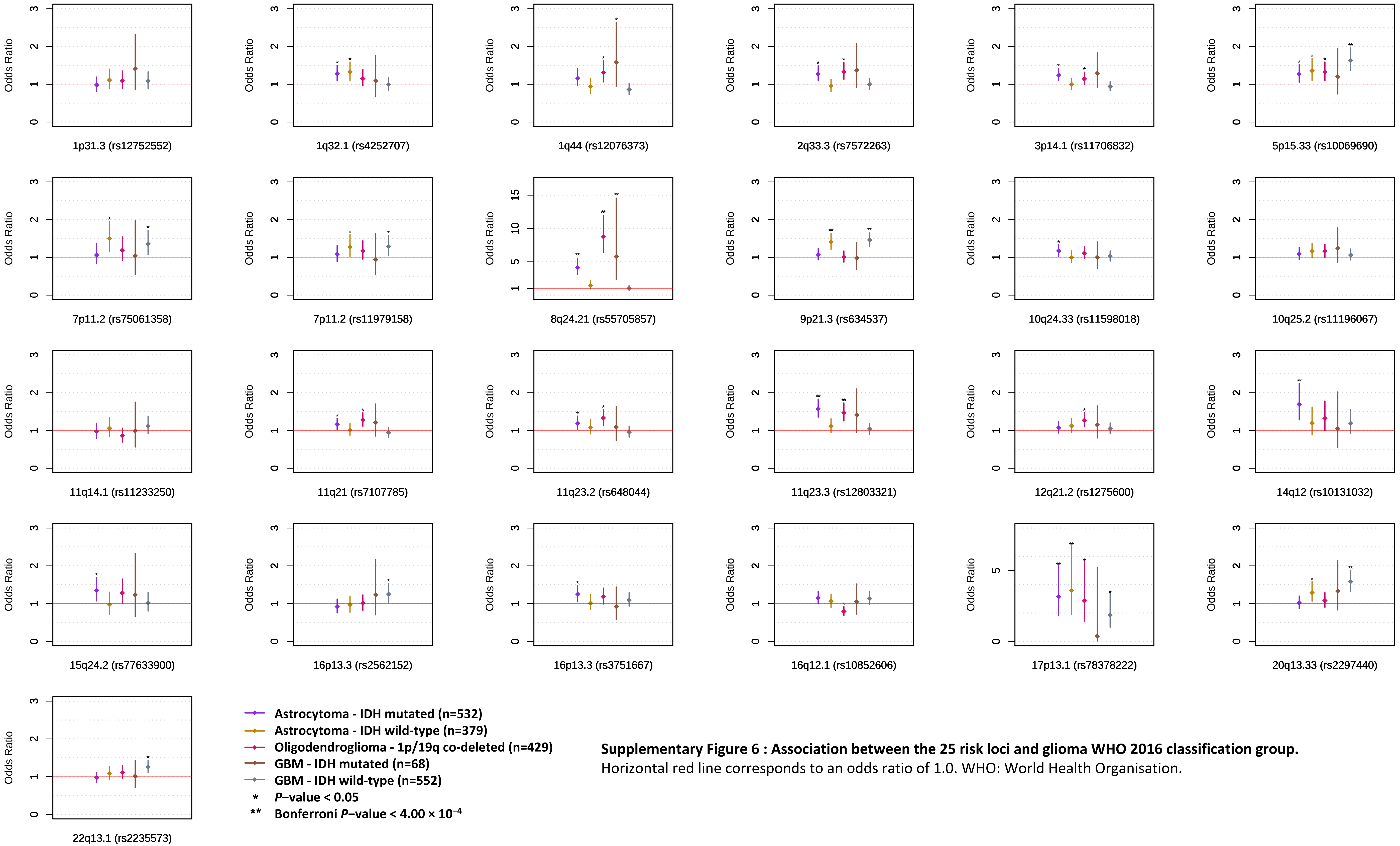

Supplementary Figure 6 : Association between the 25 risk loci and glioma WHO 2016 classification group. Horizontal red line corresponds to an odds ratio of 1.0. WHO: World Health Organisation.

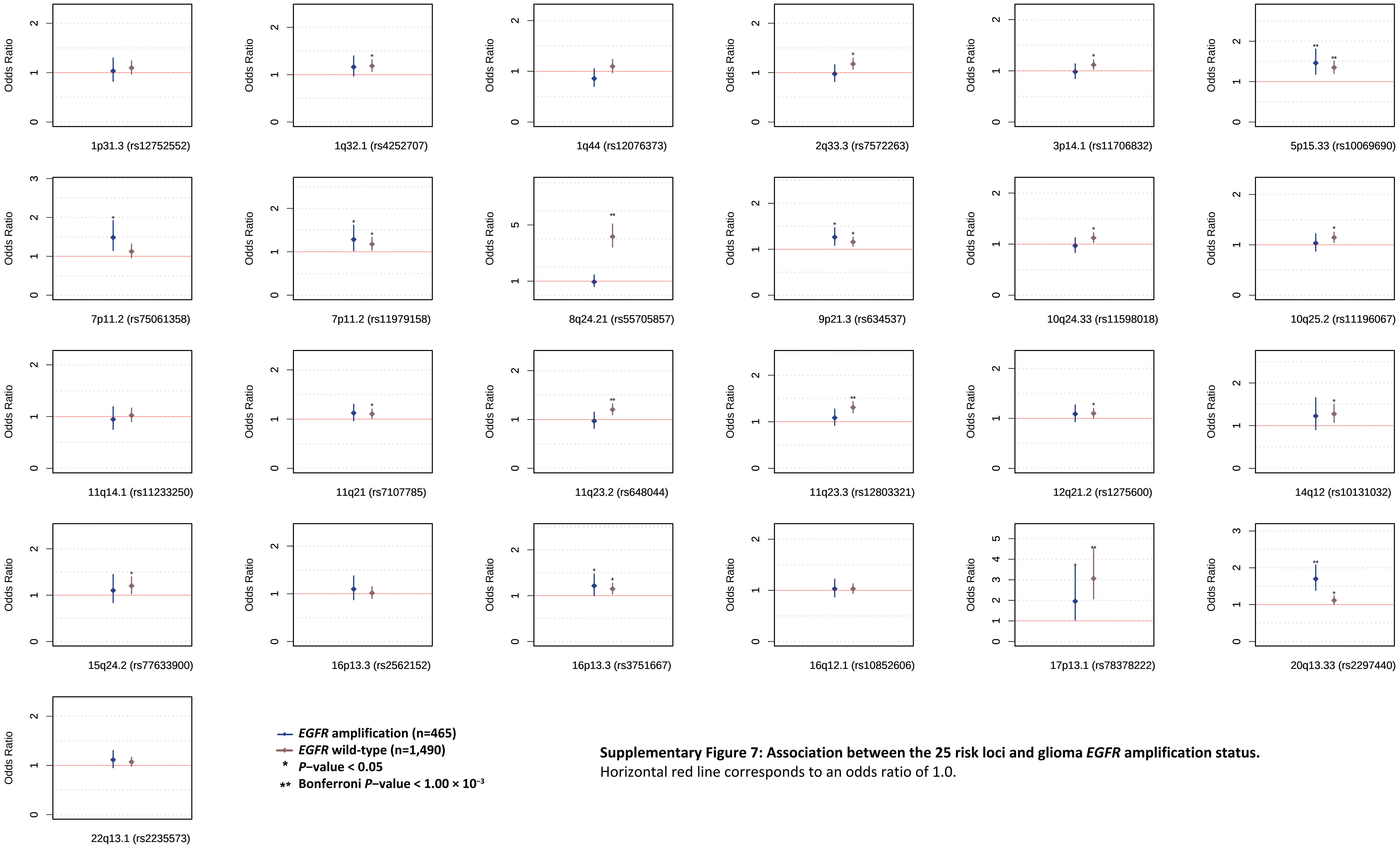

Supplementary Figure 7: Association between the 25 risk loci and glioma *EGFR* amplification status. Horizontal red line corresponds to an odds ratio of 1.0.

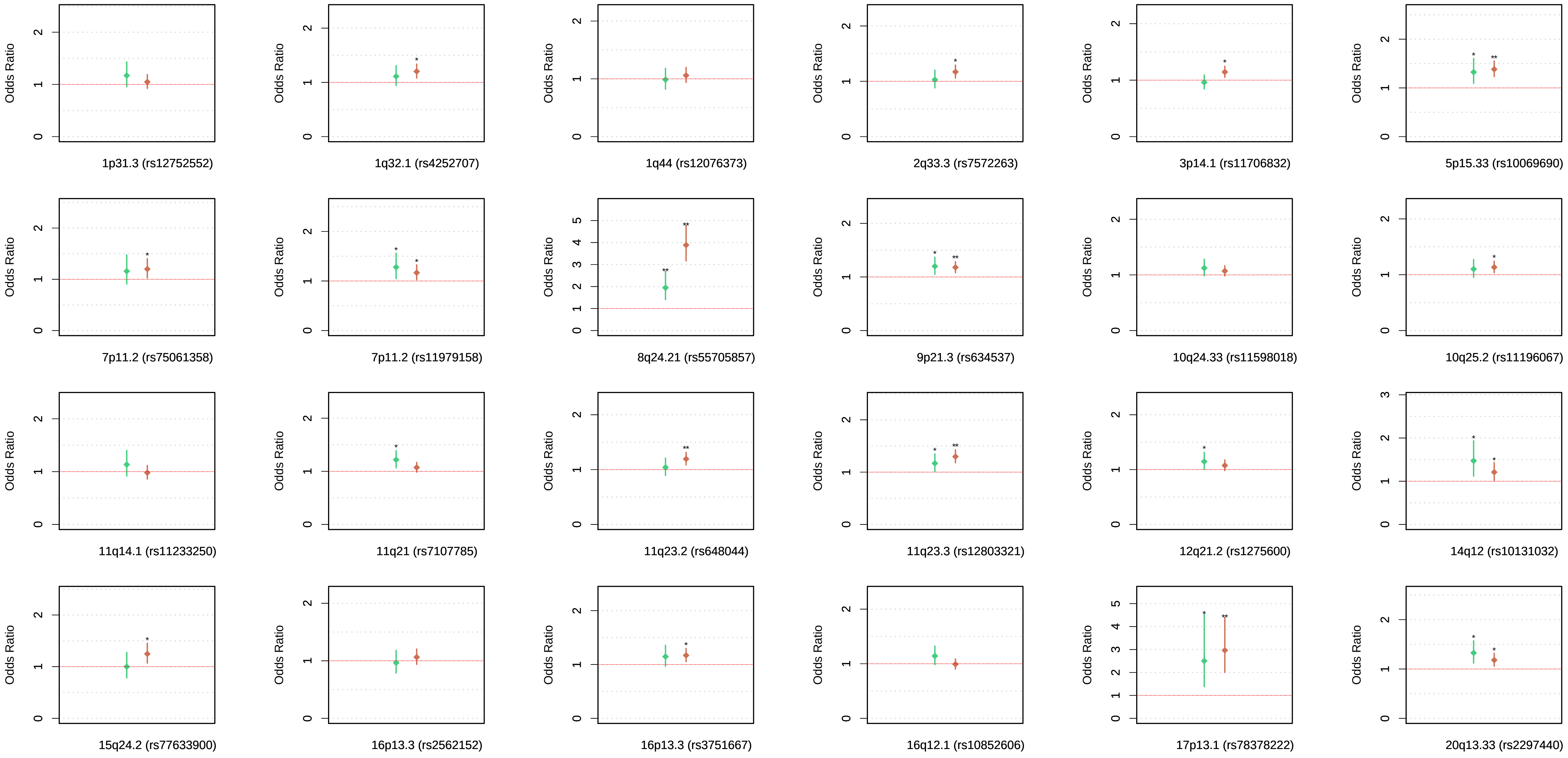

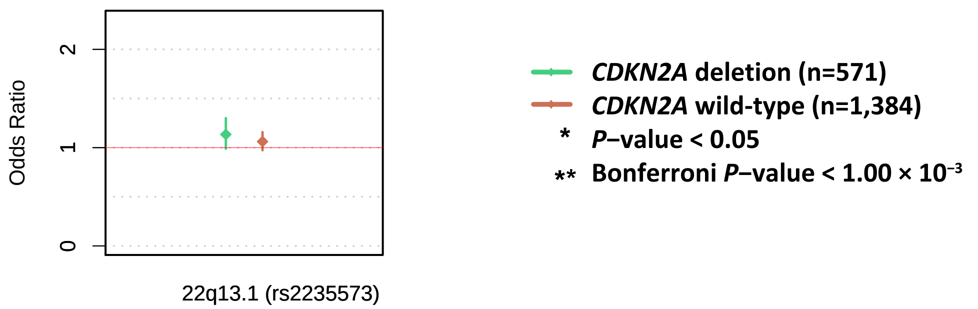

Supplementary Figure 8: Association between the 25 risk loci and glioma *CDKN2A* deletion status.
Horizontal red line corresponds to an odds ratio of 1.0.

Supplementary Figure 9: Effect of risk allele number on age at diagnosis by glioma molecular subgroup. (a) IDH-only; (b) *TERT*-only; (c) *TERT*-IDH; (d) Triple-positive; (e) Triple-negative; (f) Combined. *P*-values and correlation statistics in the top right corner are Pearson correlation statistics between risk allele number and age at diagnosis.

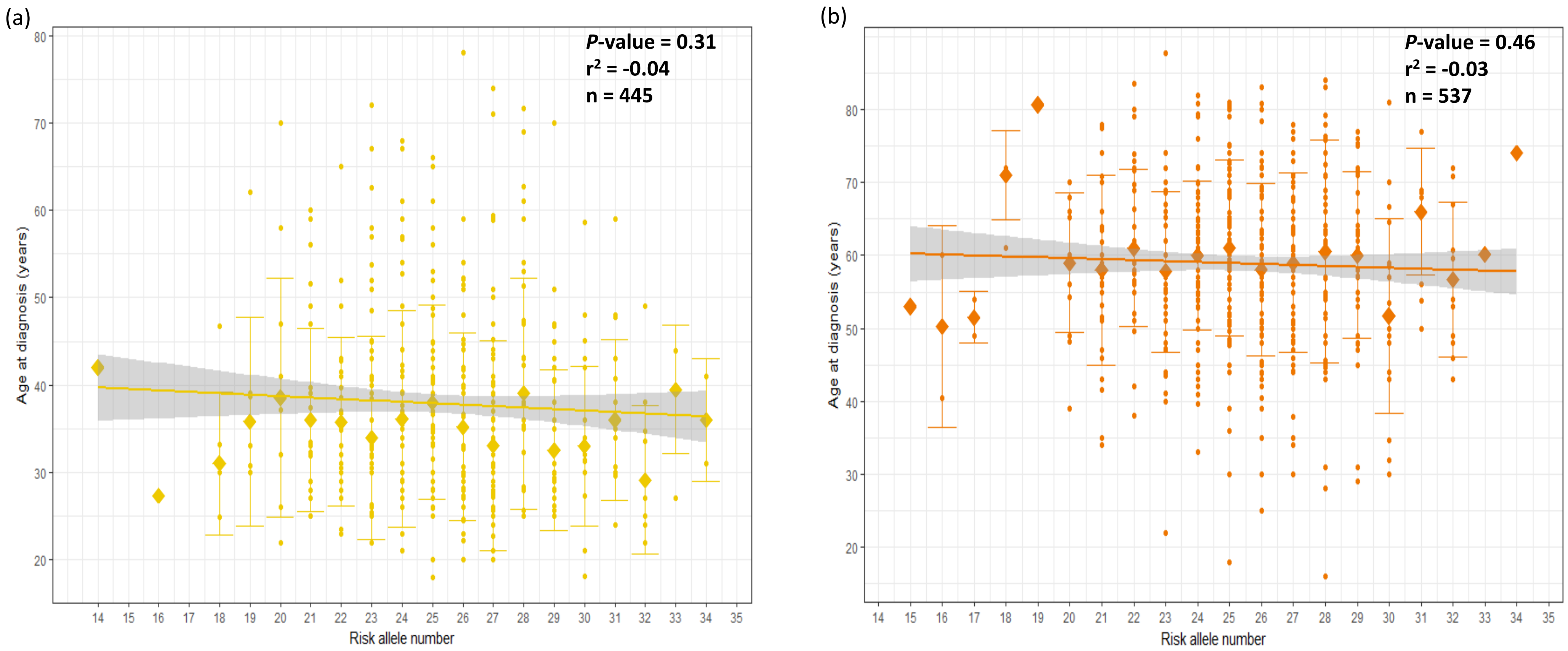

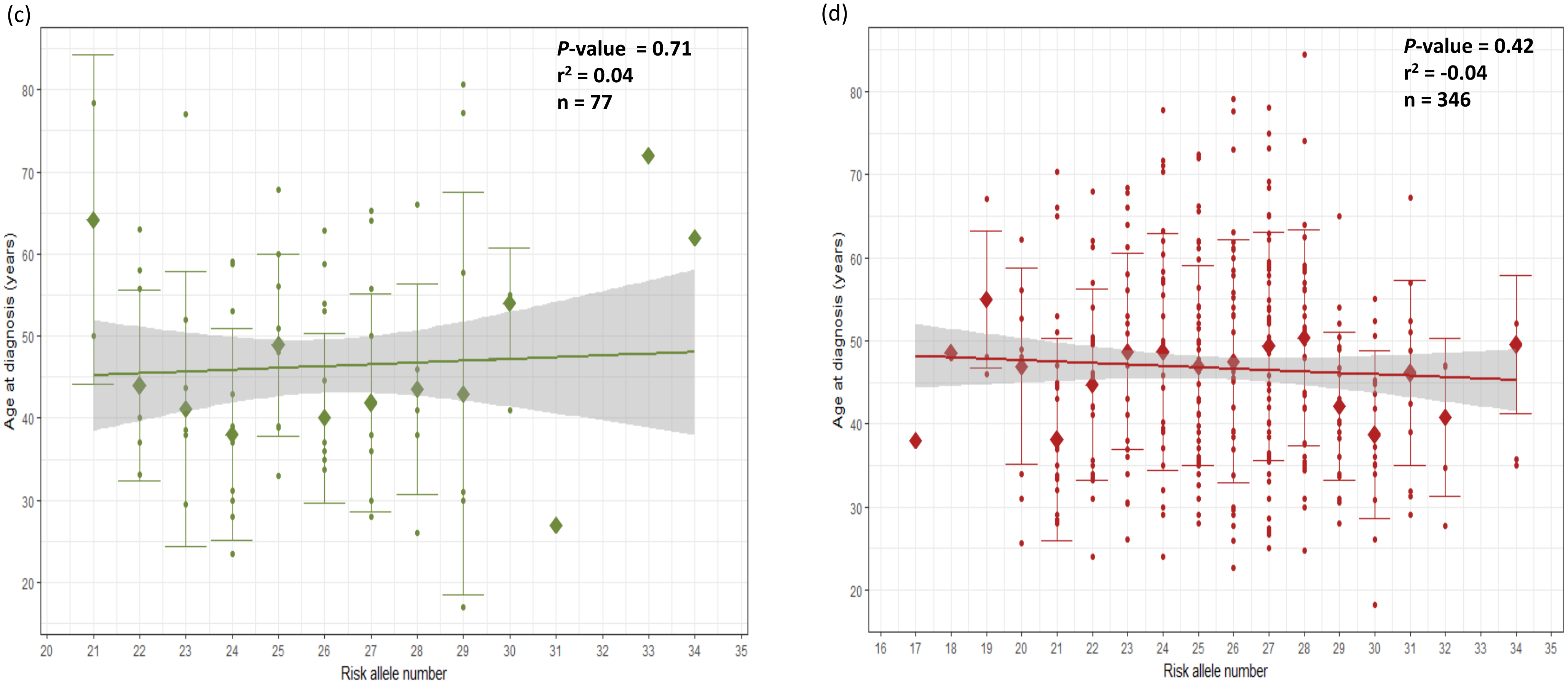

(c)
P-value = 0.71
r2 = 0.04
n = 77
Age at diagnosis (years)
Risk allele number
(d)
P-value = 0.42
r2 = -0.04
n = 346
Age at diagnosis (years)
Risk allele number

(e)

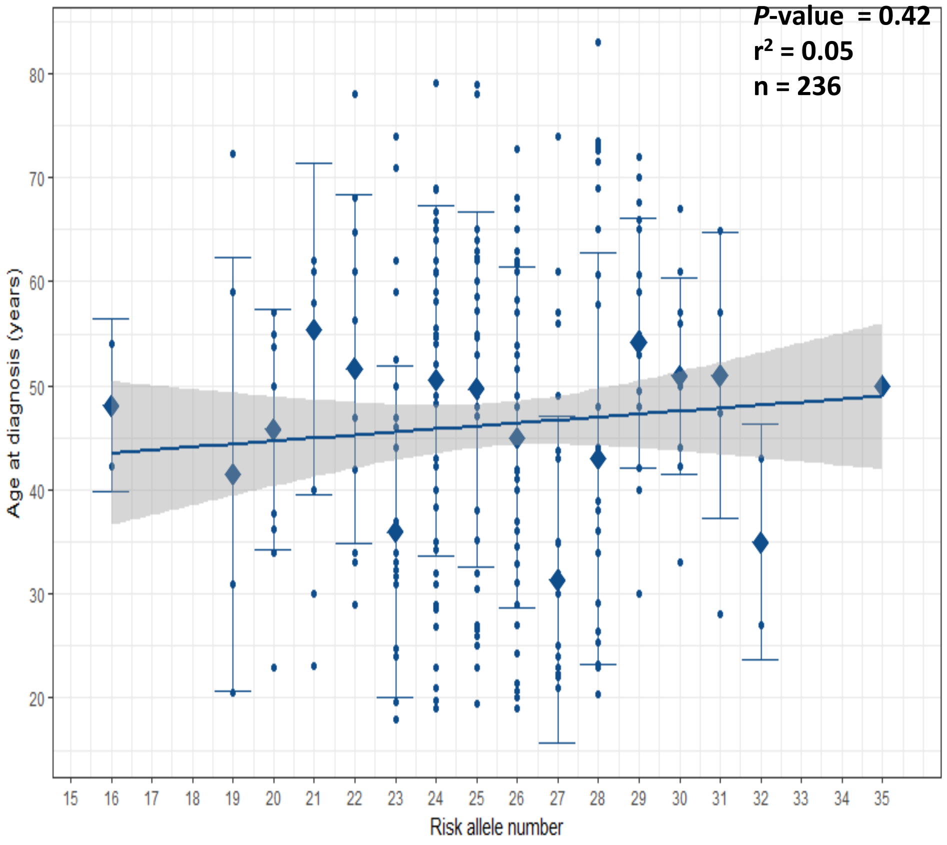
P-value = 0.42
r² = 0.05
n = 236
Age at diagnosis (years)
Risk allele number

(f)

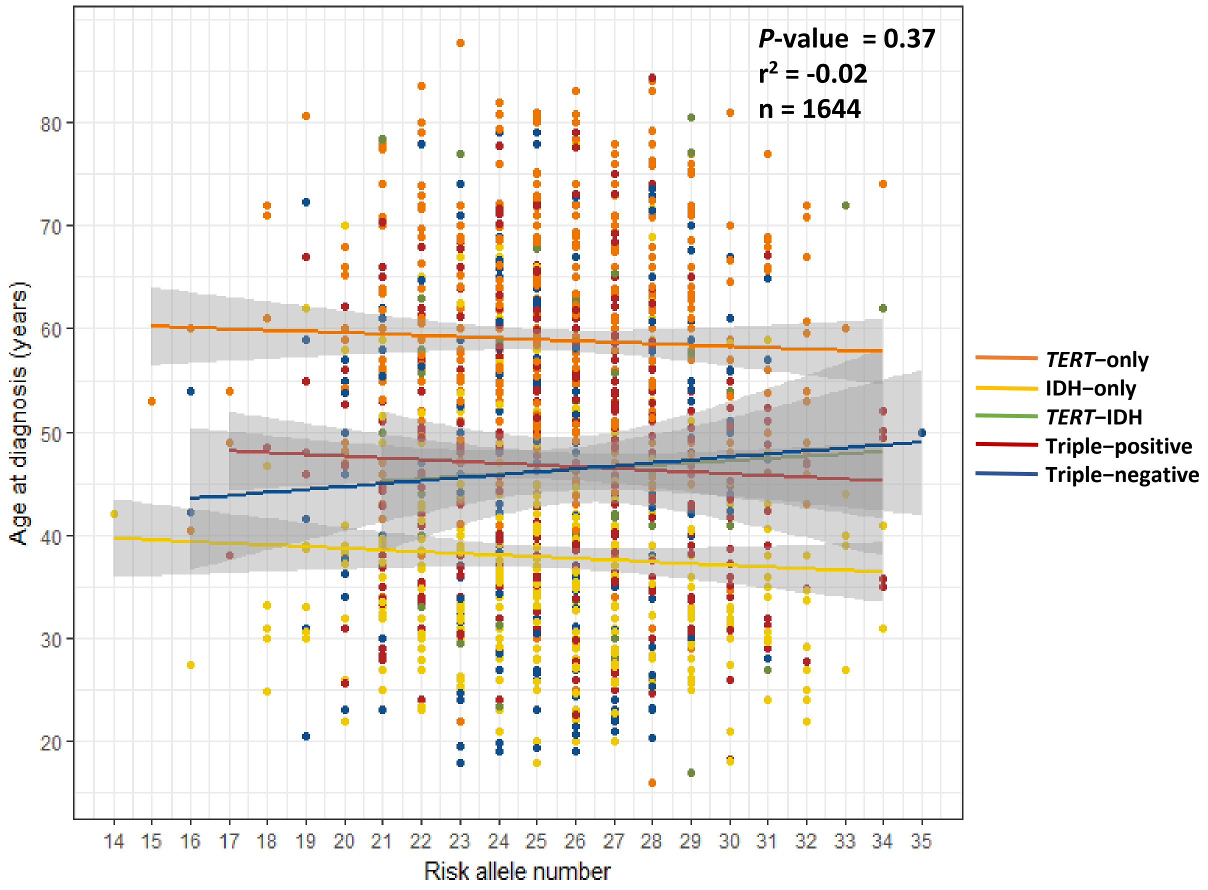
P-value = 0.37
r² = -0.02
n = 1644
Age at diagnosis (years)
Risk allele number
TERT–only
IDH–only
TERT–IDH
Triple–positive
Triple–negative

Supplementary Figure 10: Plots of Hi-C interactions in H1 neuronal progenitor cells at the 25 risk loci. Plots were generated using the HUGIn browser [2]. Each plot shows a "virtual 4C" of all Hi-C interactions with "bait" fragments overlapping the glioma risk SNP of interest (indicated by the shaded rectangle). Topologically associating domain (TAD) boundaries are plotted as filled blue rectangles. The purple dotted line represents the Bonferroni threshold, with interactions exceeding this threshold treated as statistically significant.

(a) 1p31.3 - rs1272552

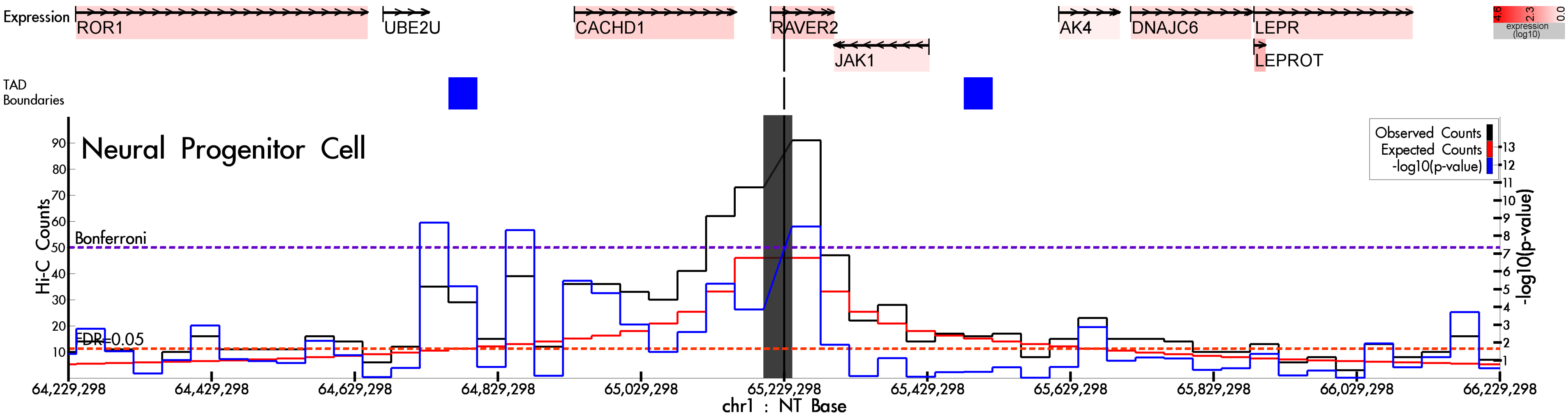

(b) 1q32.1 - rs4252707

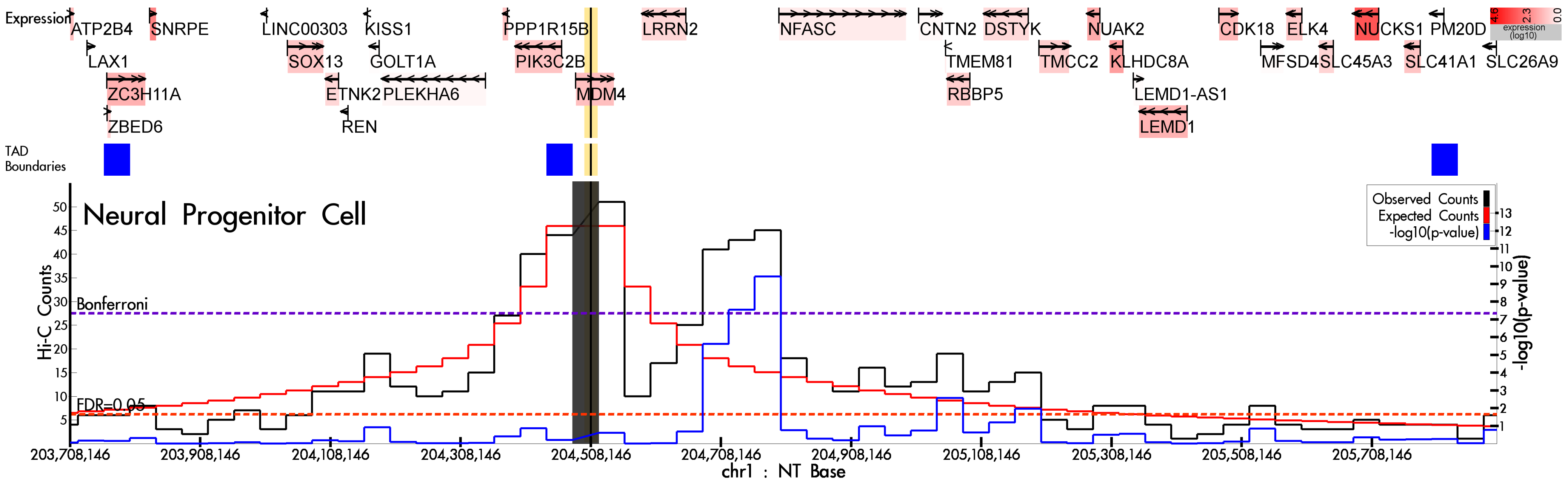

(c) 1q44 - rs12076373

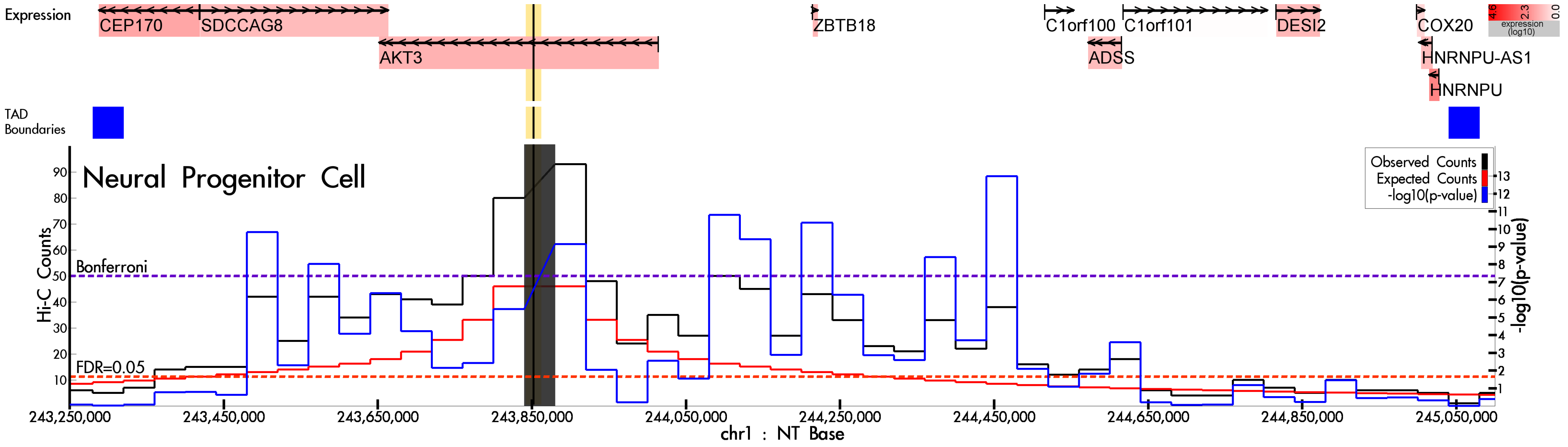

(d) 2q33.3 - rs7572263

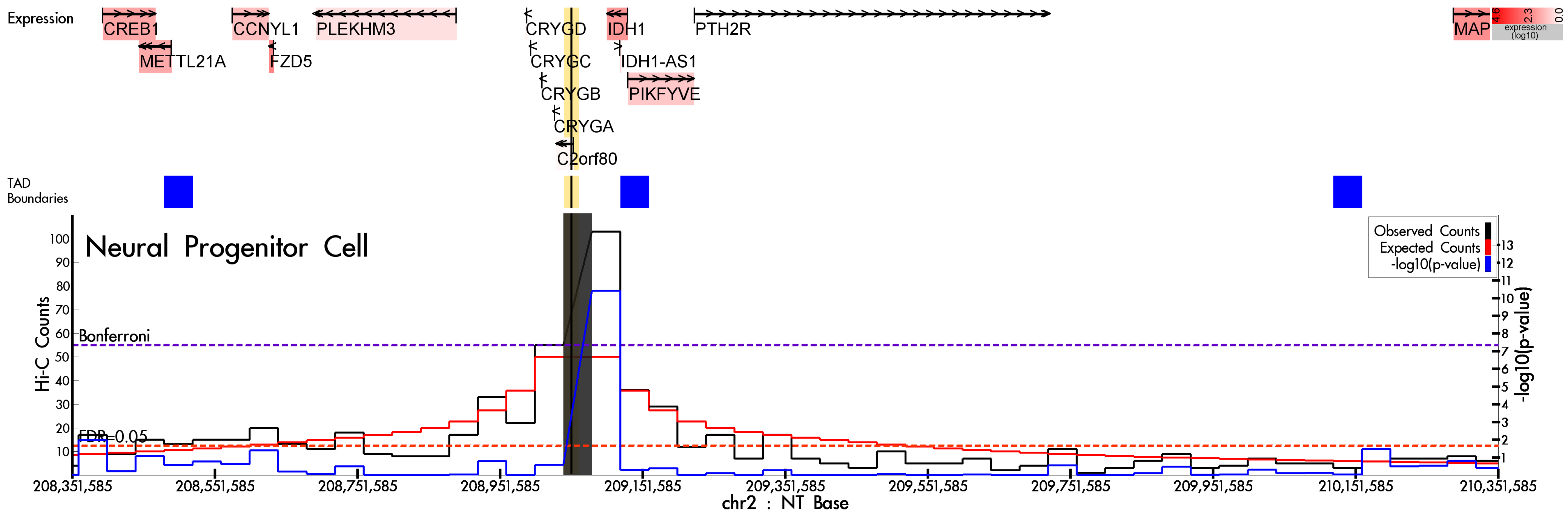

(e) 3p14.1 - rs11706832

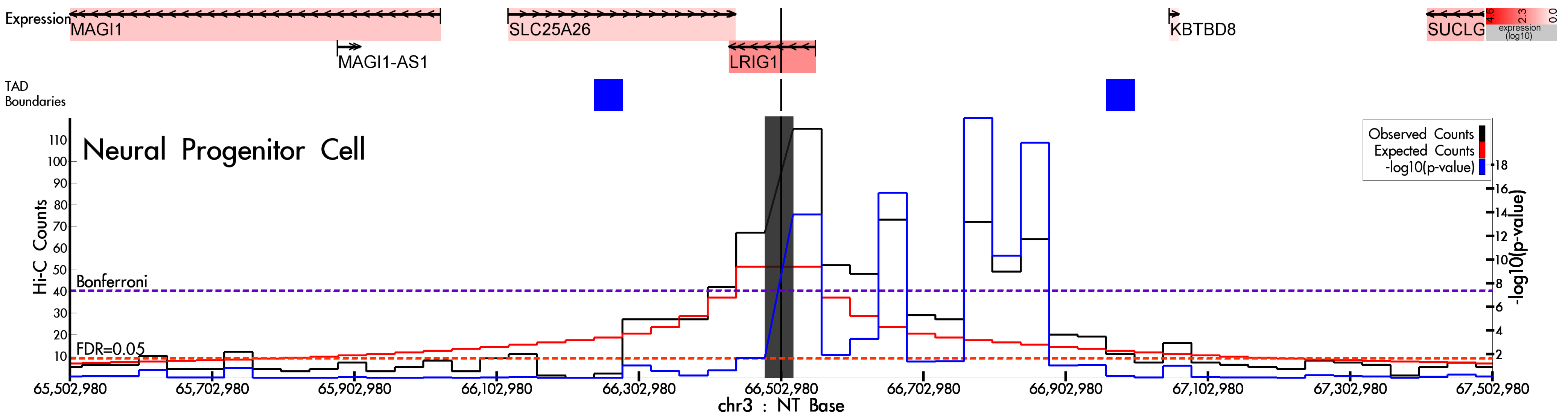

(f) 5p15.33 - rs10069690

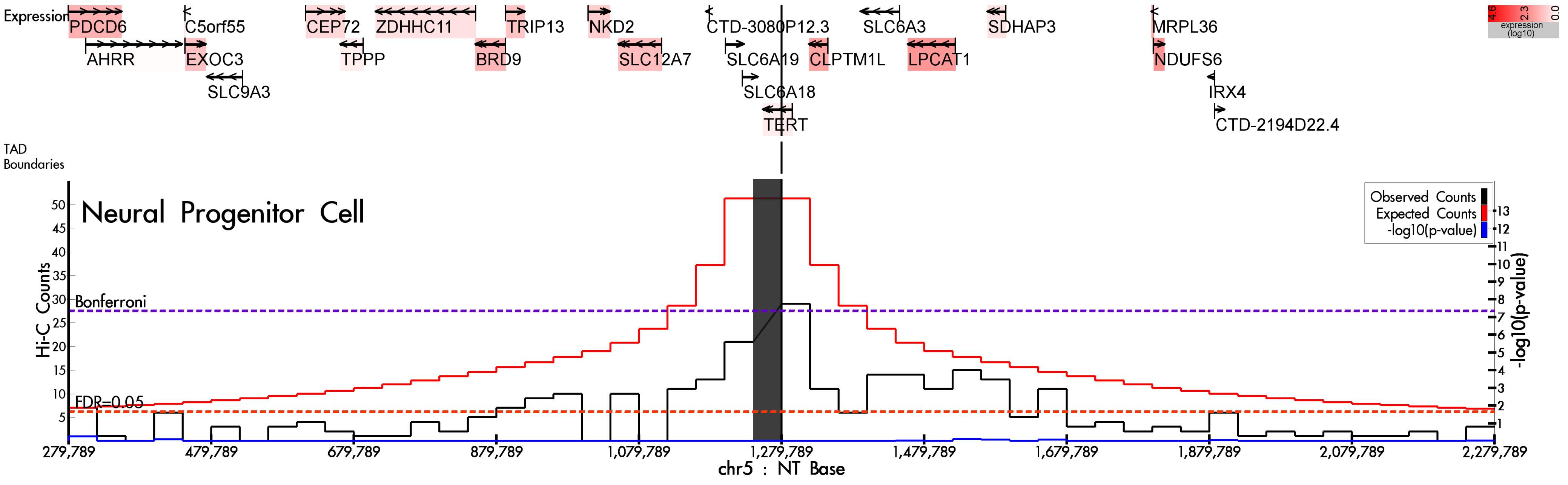

(g) 7p11.2 - rs75061358

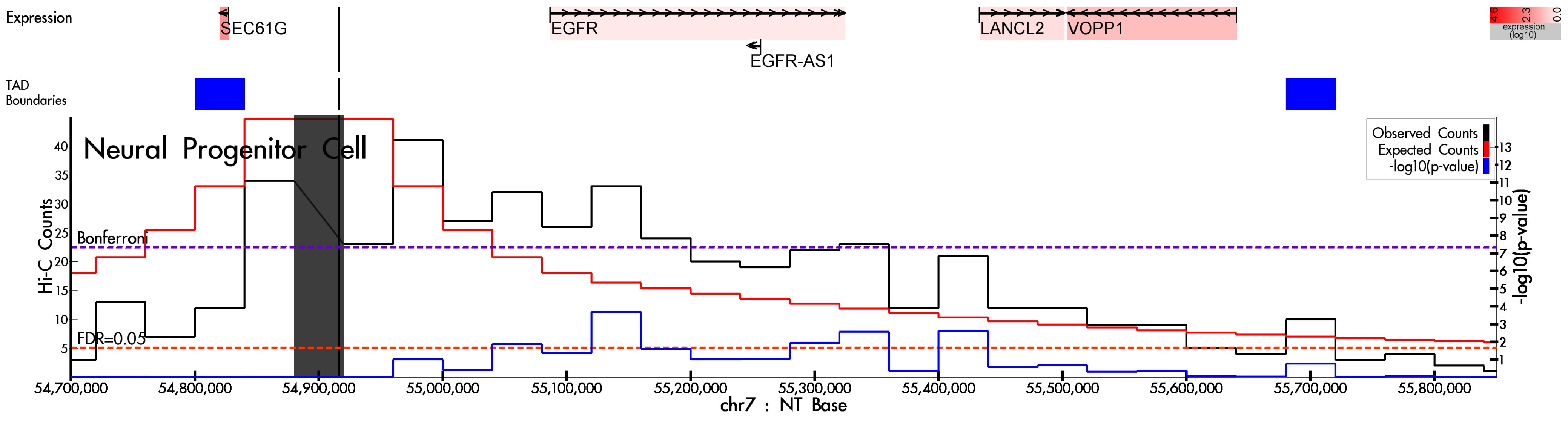

(h) 7p11.2 - rs11979158

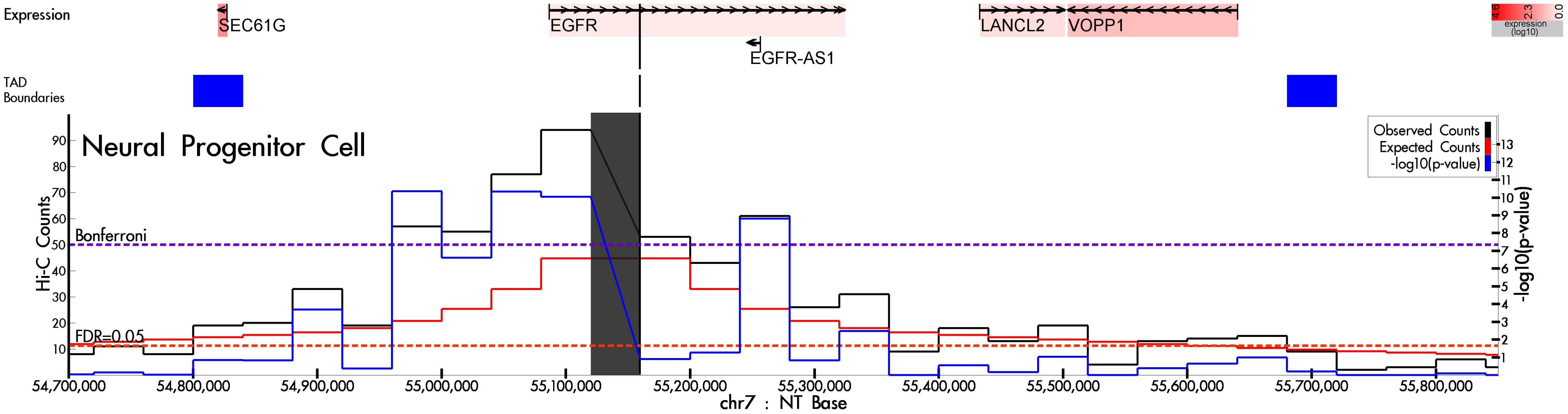

(i) 8q24.21 - rs55705857

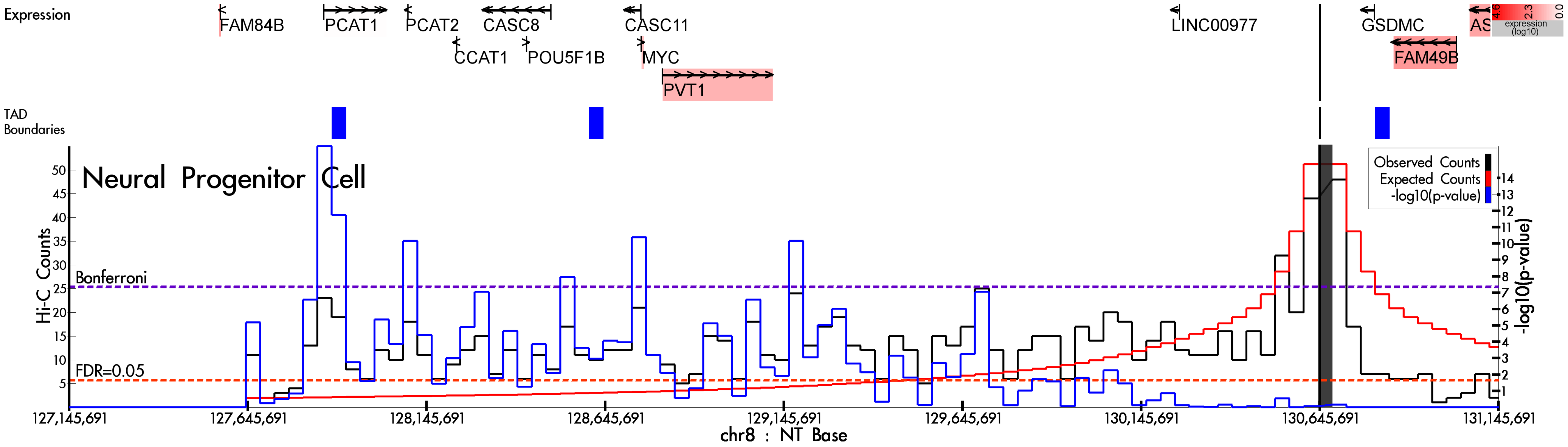

(j) 9p21.3 - rs634537

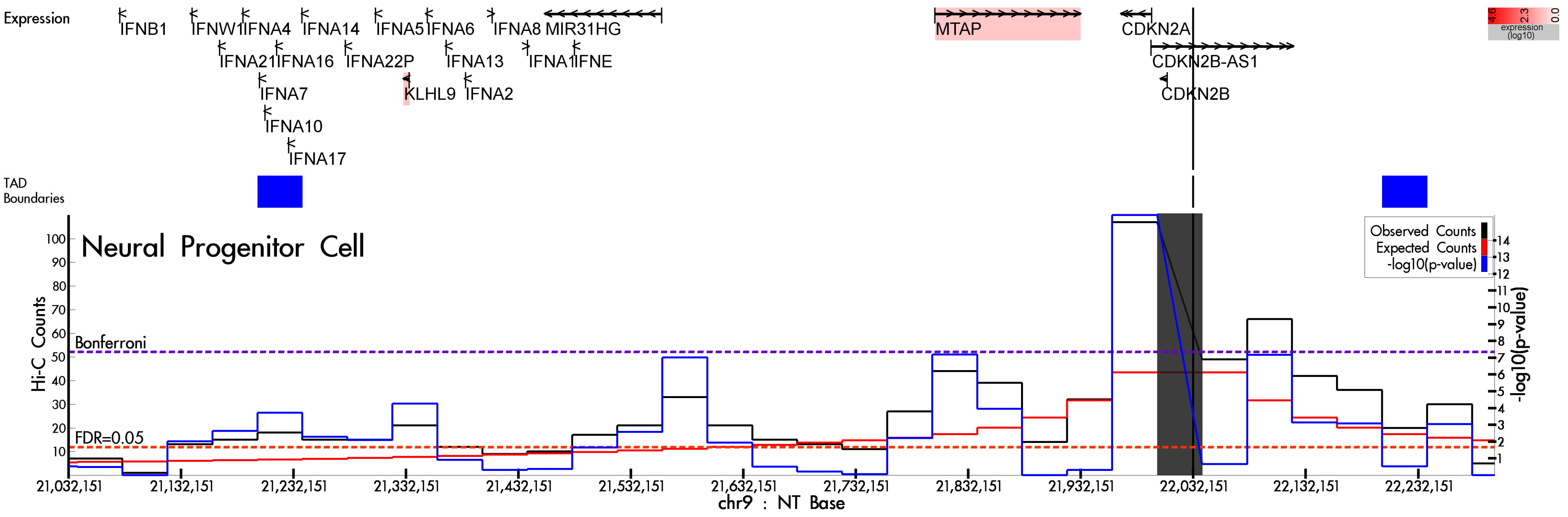

(k) 10q24.33 - rs11598018

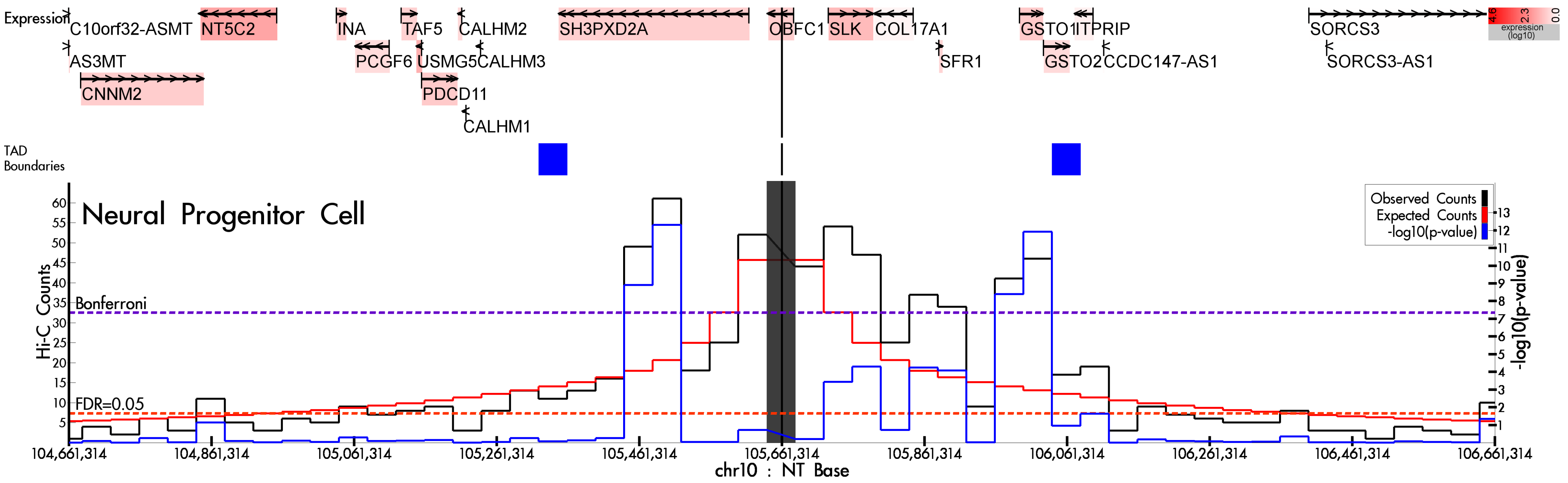

(l) 10q25.2 - rs11196067

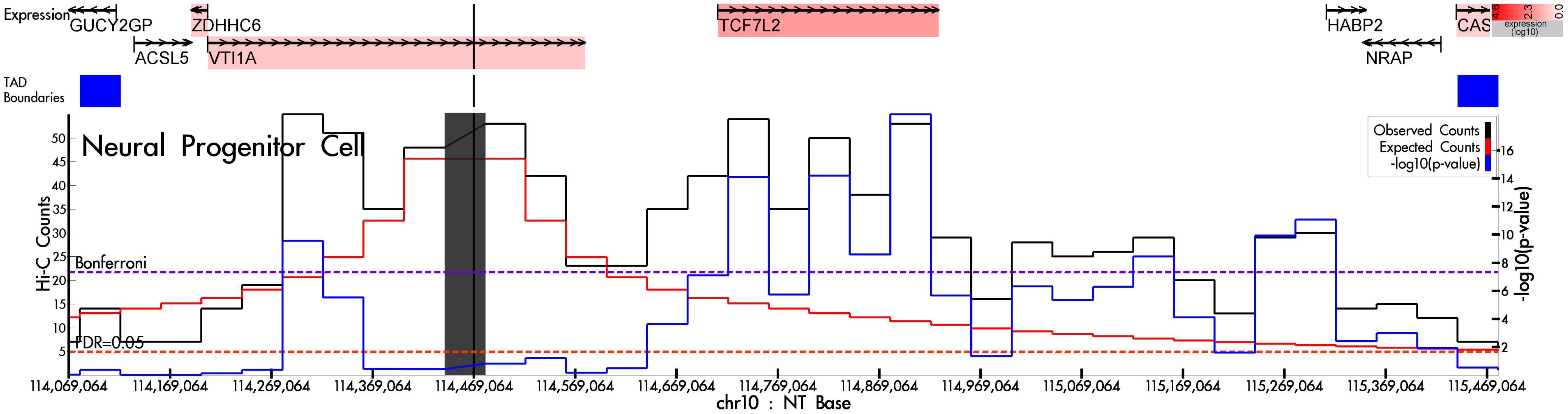

(m) 11q14.1 - rs11233250

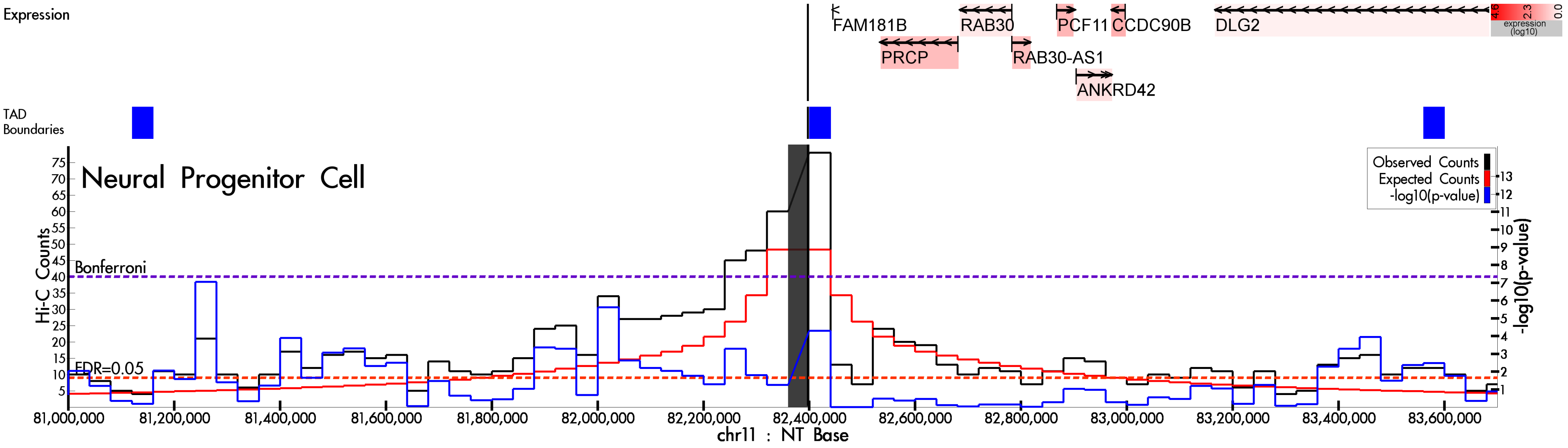

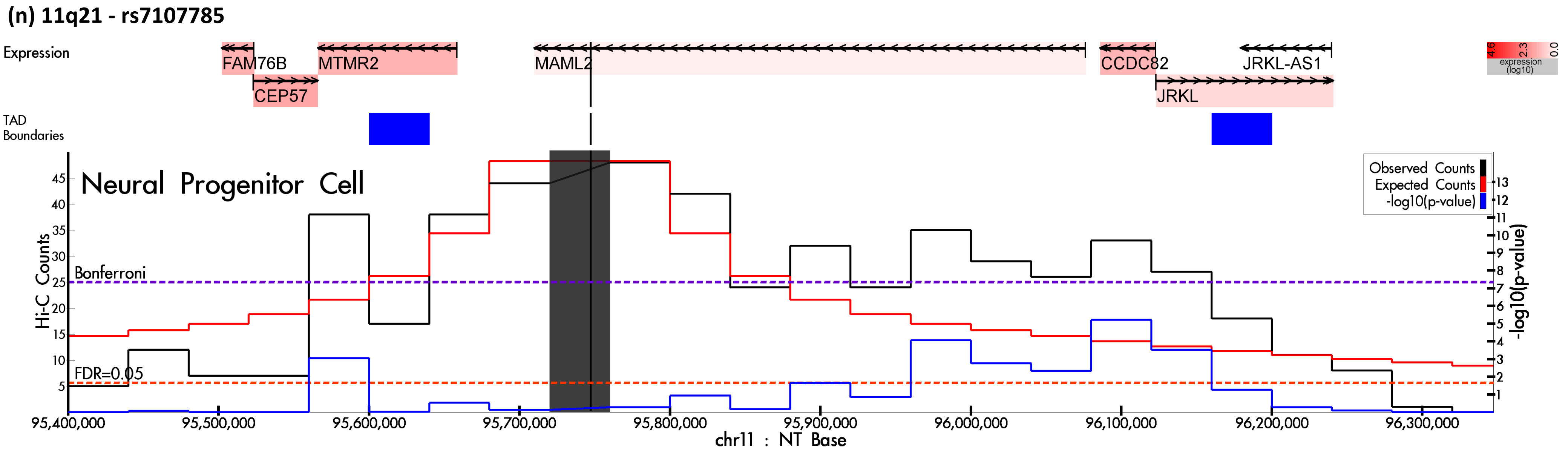
(n) 11q21 - rs7107785
Expression
FAM76B
MTMR2
MAML2
CCDC82
JRKL-AS1
CEP57
JRKL
TAD Boundaries
Neural Progenitor Cell
Observed Counts
Expected Counts
-log10(p-value)
Bonferroni
FDR=0.05
Hi-C Counts
-log10(p-value)
95,400,000
95,500,000
95,600,000
95,700,000
95,800,000
95,900,000
96,000,000
96,100,000
96,200,000
96,300,000
chr11 : NT Base

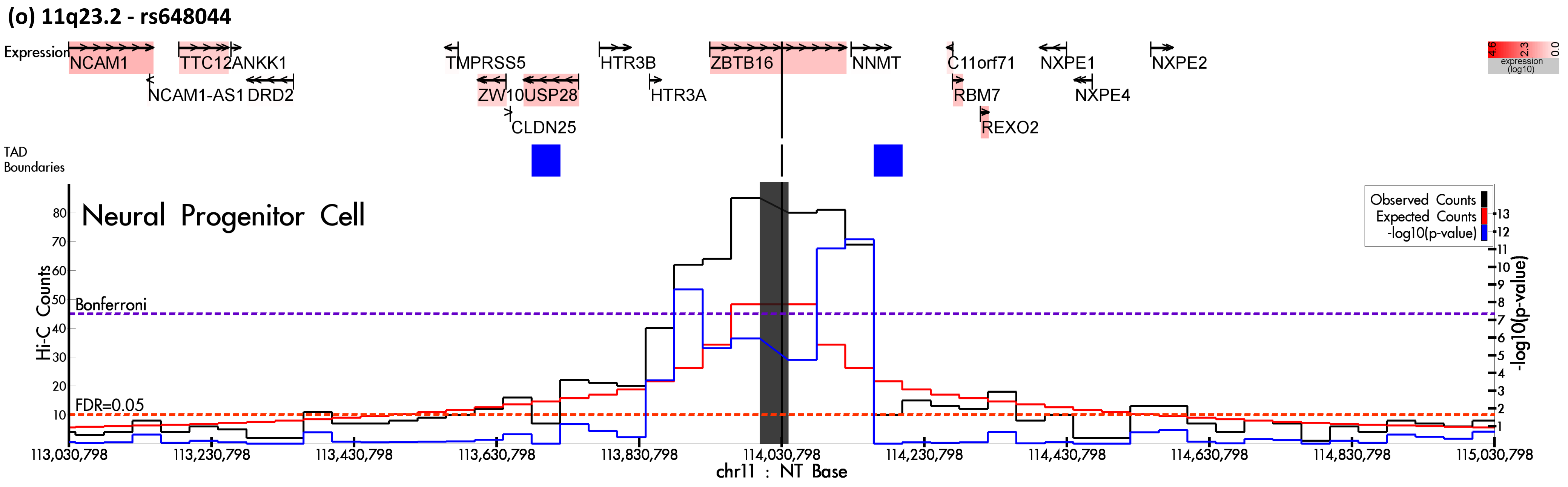
(o) 11q23.2 - rs648044
Expression
NCAM1
TTC12
ANKK1
TMPRSS5
HTR3B
ZBTB16
NNMT
C11orf71
NXPE1
NXPE2
NCAM1-AS1
DRD2
ZW10
USP28
HTR3A
RBM7
NXPE4
CLDN25
REXO2
TAD Boundaries
Neural Progenitor Cell
Observed Counts
Expected Counts
-log10(p-value)
Bonferroni
FDR=0.05
Hi-C Counts
-log10(p-value)
113,030,798
113,230,798
113,430,798
113,630,798
113,830,798
114,030,798
114,230,798
114,430,798
114,630,798
114,830,798
115,030,798
chr11 : NT Base

(p) 11q23.3 - rs12803321

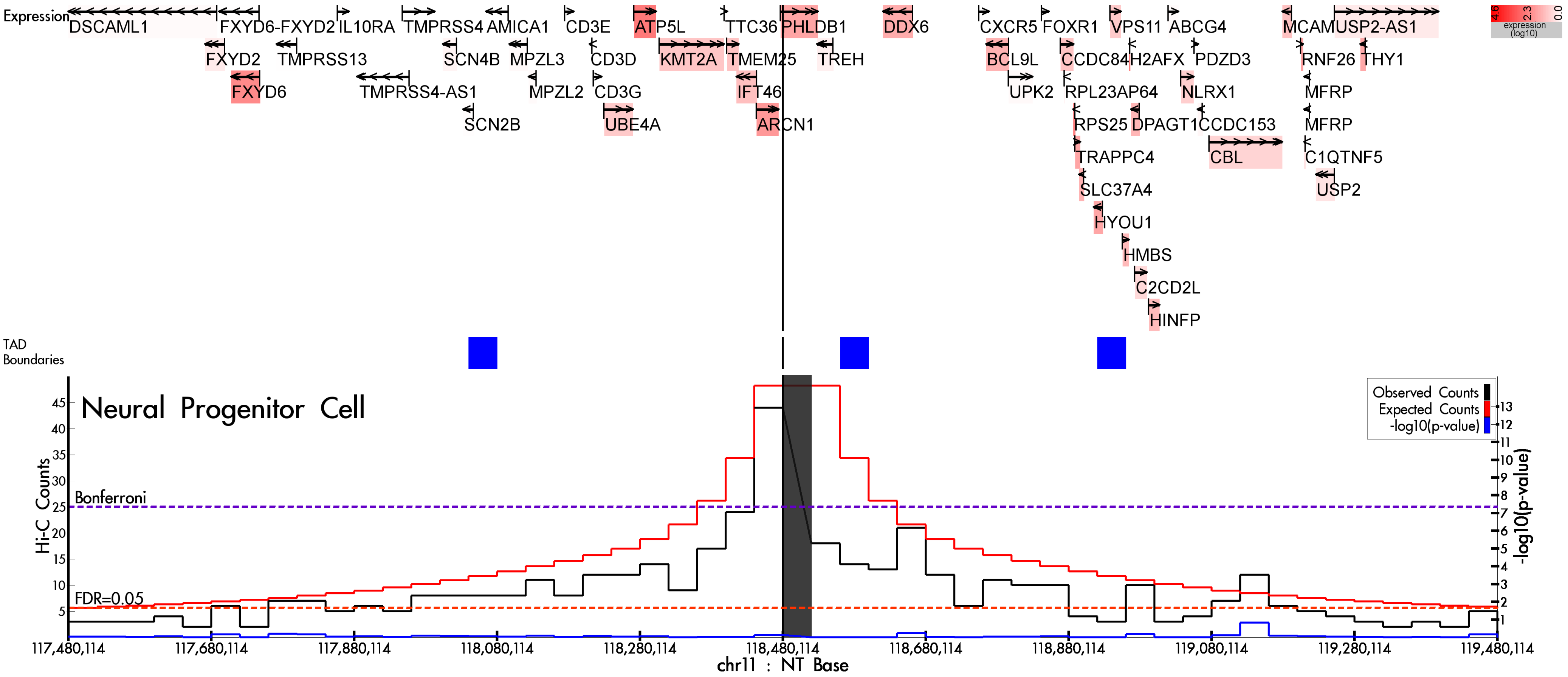

(q) 12q21.2 - rs1275600

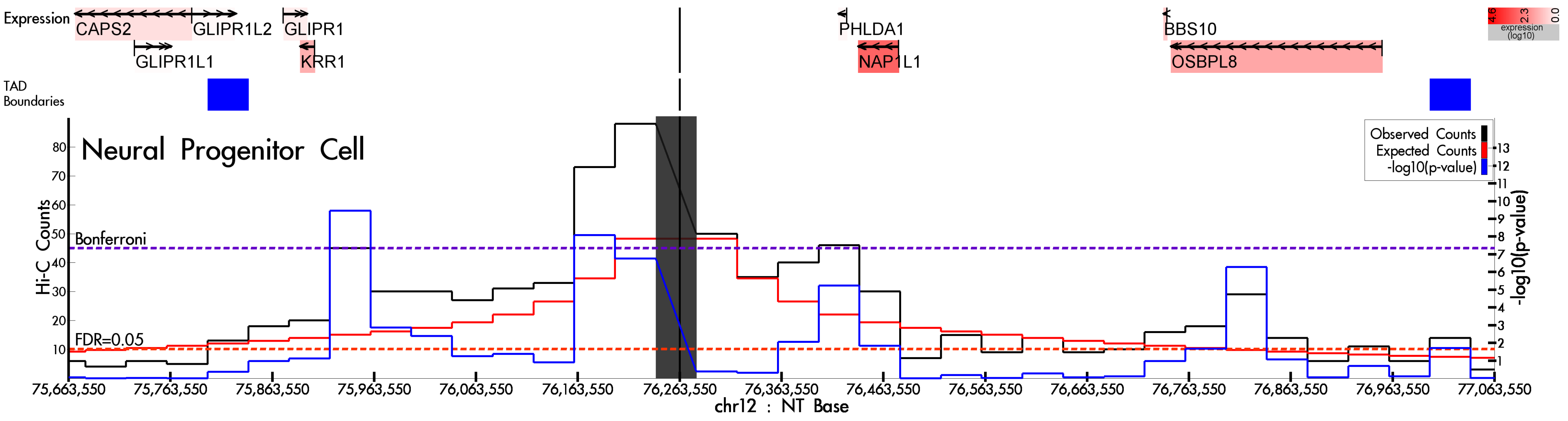

(r) 14q12 - rs10131032

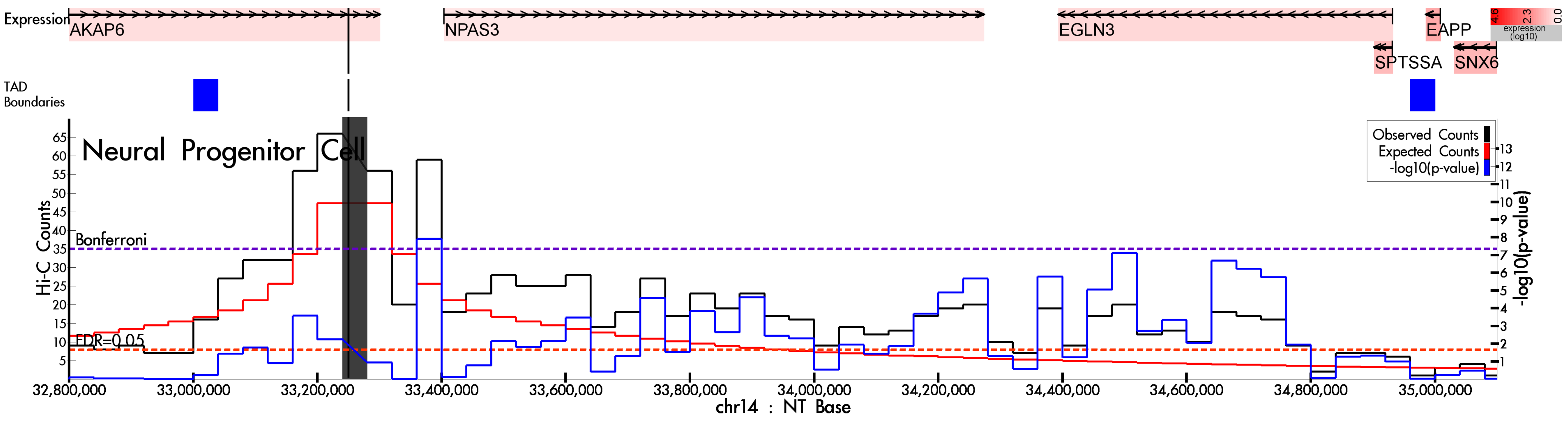

(s) 15q24.2 - rs77633900

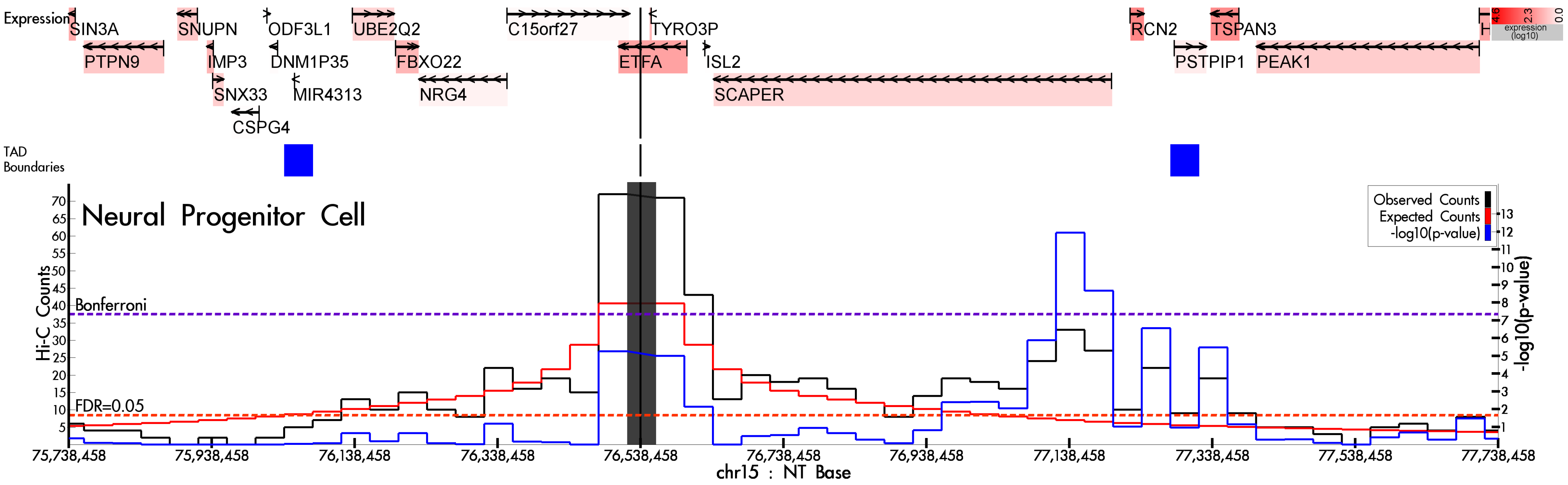

(t) 16p13.3 - rs2562152

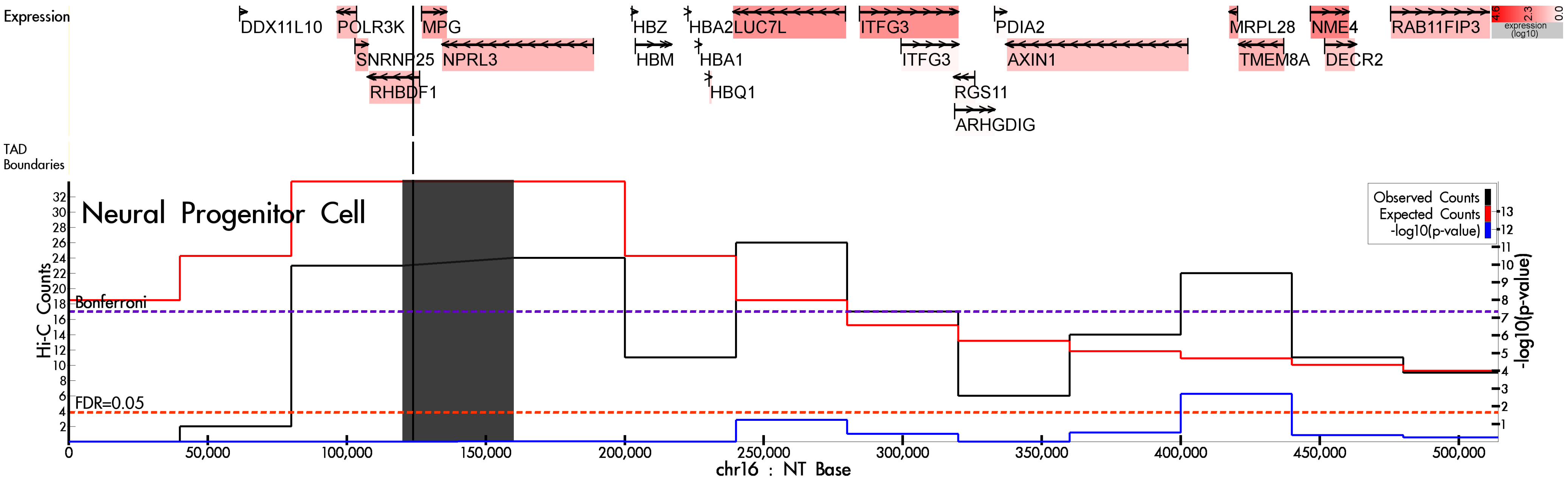

(u) 16p13.3 - rs3751667

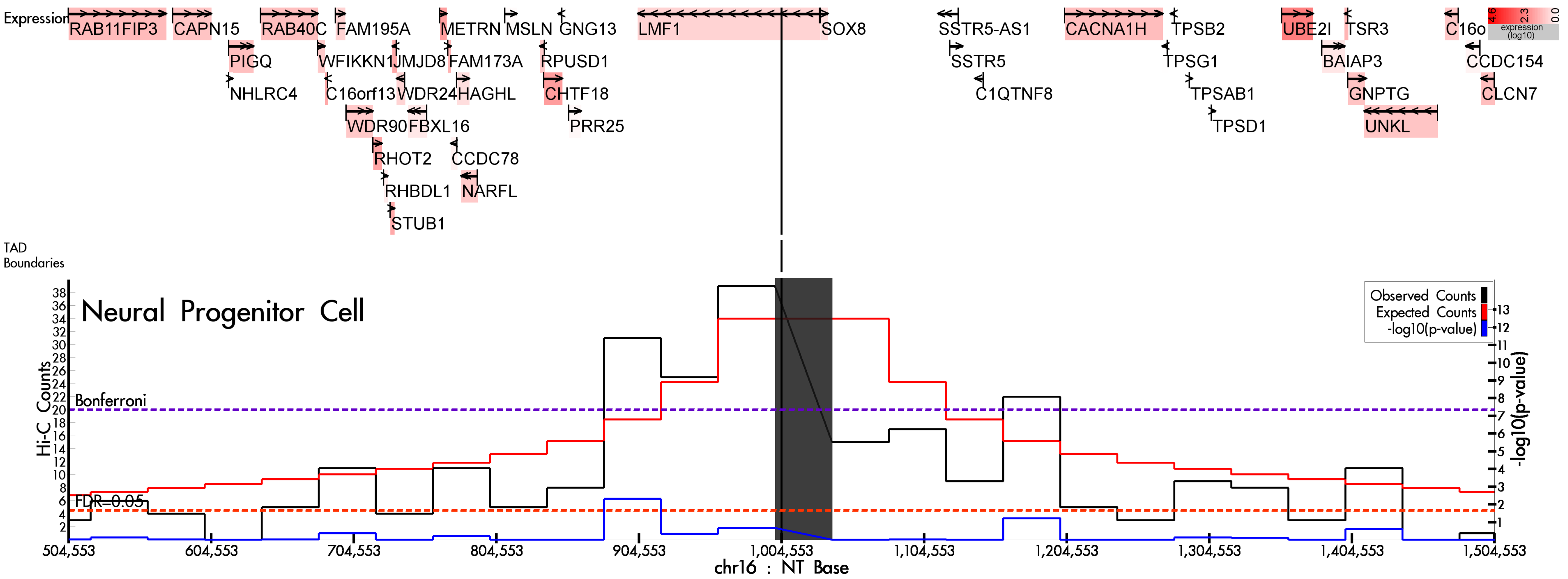

(v) 16q12.1 - rs10852606

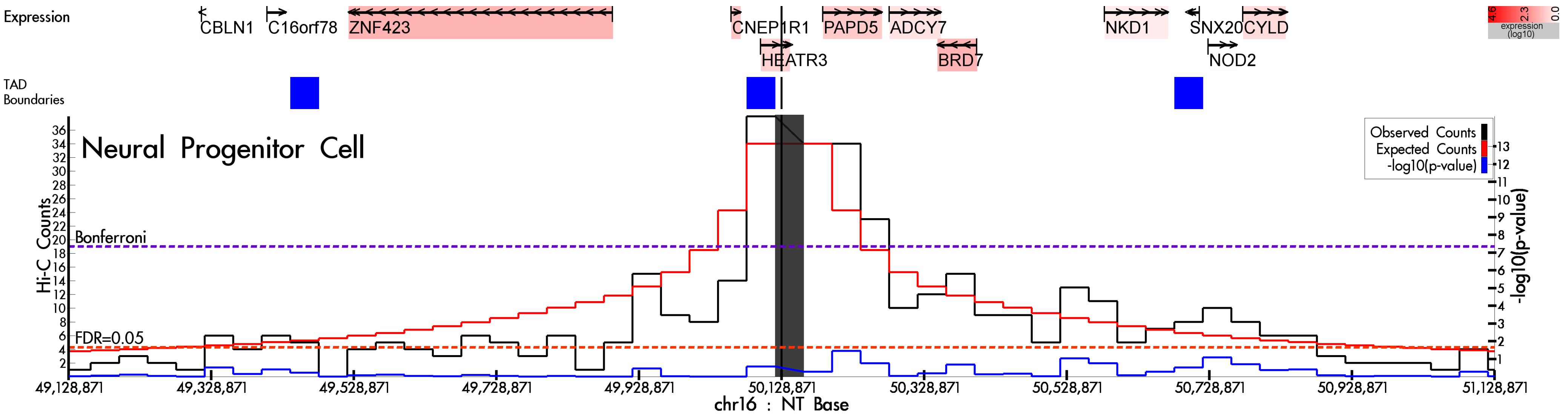

(w) 17p13.1 - rs78378222

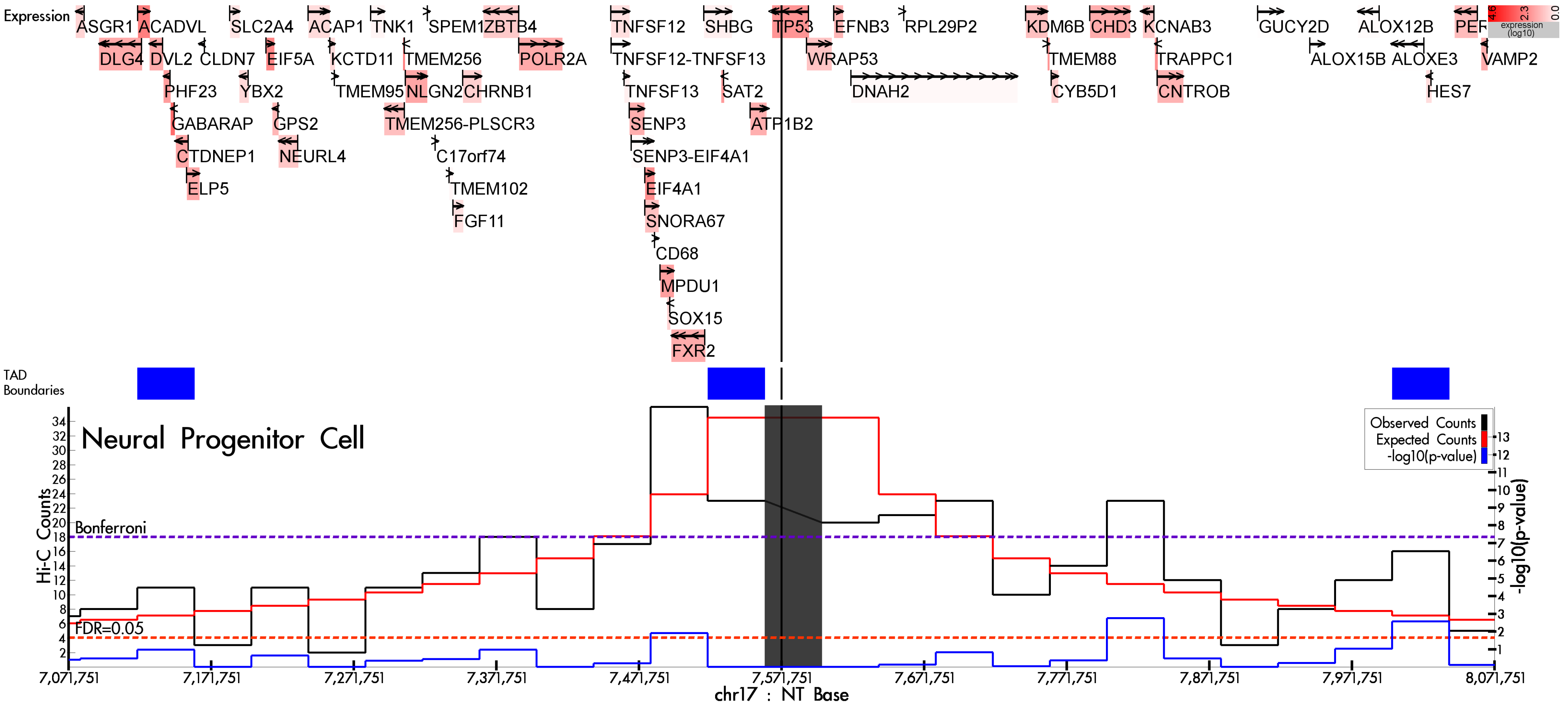

(x) 20q13.33 - rs2297440

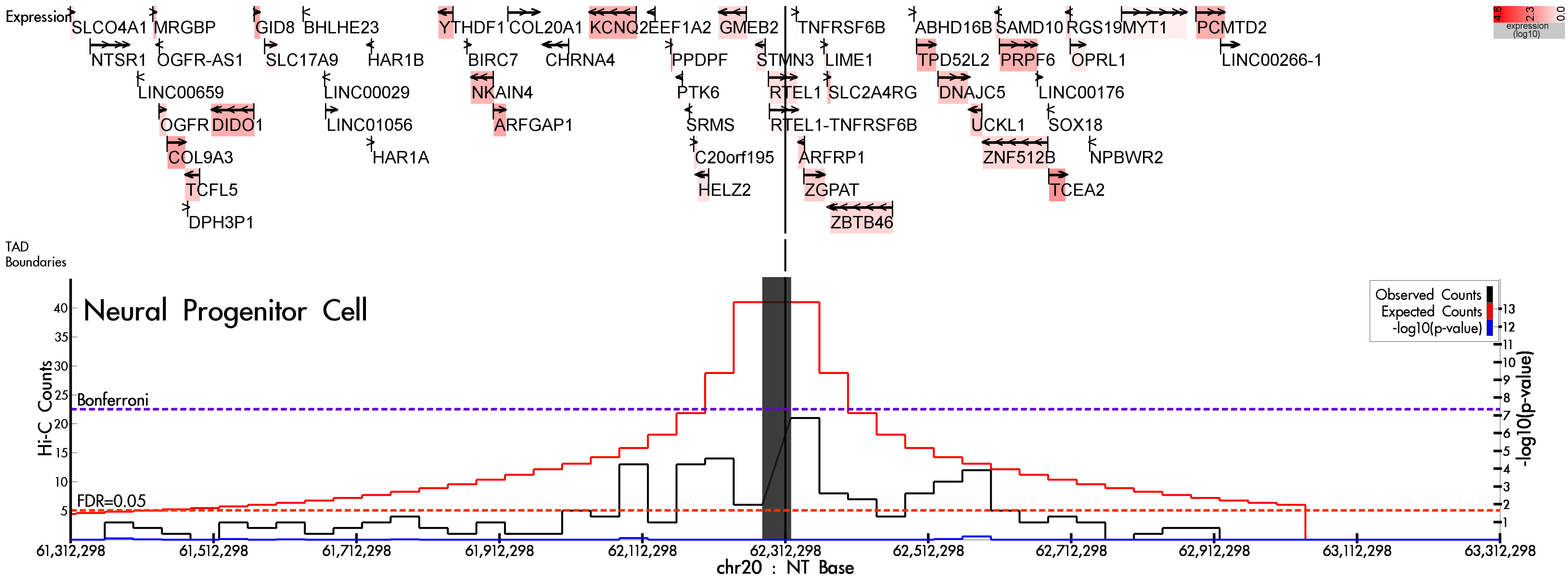

(y) 22q13.33 - rs2235573

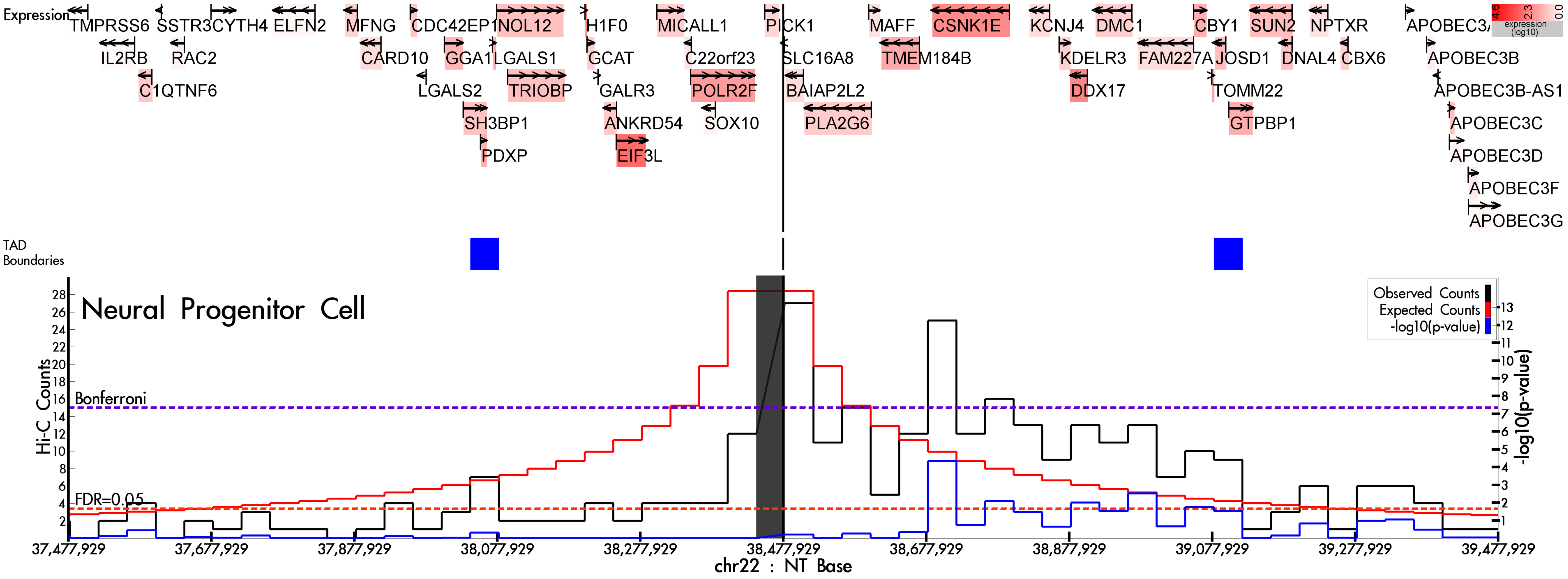

Supplementary Table 1: Details of the quality control filters applied to TCGA and French sequencing studies. Samples were excluded due to call rate (< 90% or failed genotyping), ethnicity (principal components analysis or other samples reported to be not of white, European descent), relatedness (any individuals found to be duplicated or related within or between data sets through identity by state) or sex discrepancy. TCGA, The Cancer Genome Atlas.

	**TCGA**		**French sequencing**	
	**Cases**	**Controls**	**Cases**	**Controls**
**Pre-quality control**	754	2,662	815	5,527
**Sex discrepancy**	24	-	-	-
**Call rate <0.9**	-	-	111	-
**Heterozygosity rate**	55	10	-	-
**Related Individuals**	-	5	-	-
**Non-European Ancestry**	179	-	-	-
**Post-quality control**†	521	2,648	704	5,527

† filters for quality control were performed simultaneously so numbers for each criteria may not sum to total removed.

Supplementary Table 2: Case distributions of glioma molecular subgroups by WHO 2016 classifications. Gliomas organised by molecular group were compared with those using the WHO 2016 classification [1]. Sample numbers with missing data are provided to aid comparison with Table 1.

	Molecular group							
**WHO 2016 group**	**IDH-only**	***TERT*-only**	**IDH-codel**	***TERT*-IDH**	**Triple-positive**	**Triple-negative**	**Missing**	**Total**
**Astrocytoma - IDH mutated**	404 (90%)	0	0	63 (81%)	0	0	65	532
**Astrocytoma - IDH wild-type**	0	215 (40%)	0	0	0	152 (63%)	12	379
**Oligodendroglioma - 1p/19q co-deleted**	0	0	29 (100%)	0	349 (100%)	0	51	429
**GBM - IDH mutated**	46 (10%)	0	0	15 (19%)	0	0	7	68
**GBM - IDH wild-type**	0	319 (59%)	0	0	0	78 (32%)	155	552
**Missing**	0	5 (0.9%)	0	0	0	13 (5%)	0	18
**Total**	**450**	**539**	**29**	**78**	**349**	**243**	**290**	**1978**

Supplementary Table 4: Linear regression analysis of age at diagnosis according to: (a) molecular group and risk allele number for the whole population; (b) molecular group and each individual SNP; (c) Number of risk alleles for IDH-only glioma; (d) Number of risk alleles for *TERT*-IDH glioma; (e) Number of risk alleles for Triple-positive glioma; (f) Number of risk alleles for Triple-negative glioma; g) number of risk alleles for *TERT*-only glioma. N indicates number of individuals included after exclusion of those with missing data (*i.e.* retaining imputed genotype probabilities > 0.9). SE, standard error.

**a) Overall**	**N**	**Estimate**	**SE**	**T value**	***P*-value**
**Molecular group**	1,646	4.87869	0.20131	24.23	$<2\times10^{-16}$
**Risk allele number**		-0.04402	0.10473	-0.42	0.674

**b) By SNP, adjusting by molecular group**	**N**	**Estimate**	**SE**	**T value**	***P*-value**
**1p31.3 – rs12752552**	1,601	-0.1225	0.6820	-0.18	0.858
**1q32.1 - rs4252707**	1,645	-0.1509	0.5548	-0.272	0.786
**1q44 – rs12076373**	1,630	-0.04314	0.63919	-0.067	0.946
**2q33.3 – rs7572263**	1,645	-0.1257	0.5189	-0.242	0.809
**3p14.1 – rs11706832**	1,646	-0.7076	0.4634	-1.527	0.127
**5p15.33 – rs10069690**	1,246	0.7037	0.5619	1.252	0.211
**7p11.2 – rs75061358**	1,583	0.3901	0.7875	0.495	0.62
**7p11.2 – rs11979158**	1,630	-0.2178	0.7032	-0.31	0.757
**8q24.21 – rs55705857**	1,511	-2.5951	0.9013	-2.879	**0.00404**
**9p21.3 – rs634537**	1,638	-0.01315	0.44504	-0.03	0.976
**10q24.33 – rs11598018**	1,644	0.4134	0.4562	0.906	0.365
**10q25.2 – rs11196067**	1,538	-0.3929	0.4780	-0.822	0.411
**11q14.1 – rs11233250**	1,640	-0.4332	0.6985	-0.62	0.535
**11q21 – rs7107785**	1,639	-0.4846	0.4635	-1.046	0.296
**11q23.2 – rs648044**	1,387	-0.1442	0.5040	-0.286	0.775
**11q23.3 – rs12803321**	1,567	-0.7047	0.4299	-1.639	0.101
**12q21.2 – rs1275600**	1,638	-0.4568	0.4694	-0.973	0.331
**14q12 – rs10131032**	1,641	-0.1583	0.9506	-0.167	0.868
**15q24.2 – rs77633900**	1,631	0.1261	0.8233	0.153	0.878
**16p13.3 – rs2562152**	1,566	0.8282	0.6407	1.293	0.196
**16p13.3 – rs3751667**	1,583	-0.4645	0.5412	-0.858	0.391
**16q12.1 – rs10852606**	1,638	0.7442	0.5082	1.465	0.143
**17p13.1 – rs78378222**	1,623	-0.8811	1.7794	-0.495	0.621
**20q13.33 – rs2297440**	1,622	0.9917	0.5986	1.657	0.0978
**22q13.1 – rs2235573**	1,596	0.1913	0.4556	0.42	0.675

Molecular group (median age in years)

**c) 1 - IDH-only (35.5)**	**N**	**Estimate**	**SE**	**T value**	***P*-value**
**Risk allele number**	445	-0.1387	0.1628	-0.852	0.395

**d) 2 - *TERT*-IDH (43.7)**	**N**	**Estimate**	**SE**	**T value**	***P*-value**
**Risk allele number**	77	-0.1066	0.6056	-0.176	0.86077

**e) 3 – Triple-positive (46.6)**	**N**	**Estimate**	**SE**	**T value**	***P*-value**
**Risk allele number**	346	-0.04088	0.21088	-0.194	0.846

**f) 4 – Triple-negative (47.6)**	**N**	**Estimate**	**SE**	**T value**	***P*-value**
**Risk allele number**	241	0.1128	0.3589	0.314	0.754

**f) 5 - *TERT*-only (59.2)**	**N**	**Estimate**	**SE**	**T value**	***P*-value**
**Risk allele number**	537	-0.07282	0.17747	-0.41	0.682

Supplementary Table 5: Cox Proportional-Hazards analysis of overall survival according to: (a) Age, Molecular group, grade and number of risk alleles for the whole population; (b) Age, Molecular group and each individual SNP; (c) Age, grade and number of risk alleles for Triple-positive glioma; (d) Age, grade and number of risk alleles for IDH-only and *TERT*-IDH glioma; (e) Age, grade and number of risk alleles for Triple-negative glioma; (f) Age, grade and number of risk alleles for *TERT*-only glioma. Due to smaller sample number of *TERT*-IDH and similarity in survival with IDH-only tumours, these were combined into one group. N indicates number of individuals included after exclusion of those with missing data (*i.e.* retaining imputed genotype probabilities > 0.9).

a) Overall	N	Coef	Exp(Coef)	Se(Coef)	Z	*P*-value
**Molecular group**	1,365	0.55223	1.73713	0.04638	11.91	**$<2x10^{-16}$**
**Age**		0.02725	1.02762	0.00333	8.18	**$3.3x10^{-16}$**
**Grade**		0.50137	1.65097	0.06152	8.15	**3.3e-16**
**Risk allele number**		-0.04396	0.95699	0.0135	-3.24	**0.0012**

b) By SNP, adjusting by molecular group	N	Coef	Exp(Coef)	Se(Coef)	Z	*P*-value
**1p31.3 – rs12752552**	1,324	-0.09958	0.90522	0.08118	-1.23	0.22
**1q32.1 - rs4252707**	1,364	-0.18006	0.83522	0.07154	-2.52	**0.012**
**1q44 – rs12076373**	1,349	0.04794	1.04911	0.07913	0.61	0.54
**2q33.3 – rs7572263**	1,364	-0.01941	0.98078	0.06488	-0.3	0.76
**3p14.1 – rs11706832**	1,365	-0.06859	0.93371	0.05540	-1.24	0.22
**5p15.33 – rs10069690**	965	-0.0757	0.9271	0.0750	-1.01	0.31
**7p11.2 – rs75061358**	1,302	-0.14607	0.86410	0.09925	-1.47	0.14
**7p11.2 – rs11979158**	1,349	-0.06373	0.93826	0.08612	-0.74	0.46
**8q24.21 – rs55705857**	1,230	-0.01939	0.98080	0.14485	-0.13	0.89
**9p21.3 – rs634537**	1,357	-0.04579	0.95524	0.05588	-0.82	0.41
**10q24.33 – rs11598018**	1,363	-0.01217	0.98790	0.05883	-0.21	0.84
**10q25.2 – rs11196067**	1,257	-0.07980	0.92331	0.05880	-1.36	0.17
**11q14.1 – rs11233250**	1,359	0.04696	1.04808	0.08913	0.53	0.6
**11q21 – rs7107785**	1,358	-0.13690	0.87206	0.05736	-2.39	**0.017**
**11q23.2 – rs648044**	1,106	-0.02746	0.97291	0.06413	-0.43	0.67
**11q23.3 – rs12803321**	1,298	-0.13159	0.87670	0.05440	-2.42	**0.016**
**12q21.2 – rs1275600**	1,357	-0.09904	0.90570	0.05633	-1.76	0.079
**14q12 – rs10131032**	1,360	0.23582	1.26595	0.12111	1.95	0.052
**15q24.2 – rs77633900**	1,353	0.04699	1.04811	0.10202	0.46	0.65
**16p13.3 – rs2562152**	1,285	0.0512	1.0525	0.0793	0.65	0.52
**16p13.3 – rs3751667**	1,302	-0.00969	0.99036	0.06747	-0.14	0.89
**16q12.1 – rs10852606**	1,358	0.01386	1.01396	0.06354	0.22	0.83
**17p13.1 – rs78378222**	1,342	0.11066	1.11702	0.21050	0.53	0.6
**20q13.33 – rs2297440**	1,342	-0.01284	0.98725	0.07541	-0.17	0.86
**22q13.1 – rs2235573**	1,316	-0.1039	0.9013	0.0564	-1.84	0.066

By molecular group

c) Triple-positive	N	Coef	Exp(Coef)	Se(Coef)	Z	*P*-value
**Age**	254	0.0743	1.0770	0.0177	4.20	**$1.7x10^{-5}$**
**Grade**		-0.0091	0.9909	0.3581	-0.03	0.980
**Risk allele number**		-0.1103	0.8955	0.0568	-1.94	**0.05**

d) IDH-only and *TERT*-IDH	N	Coef	Exp(Coef)	Se(Coef)	Z	*P*-value
**Age**	436	0.02682	1.02718	0.00709	43.78	**0.00016**
**Grade**		0.45560	1.57711	0.11719	3.89	**0.00010**
**Risk allele number**		-0.01522	0.98489	0.02655	-0.57	0.56640

e) Triple-negative	N	Coef	Exp(Coef)	Se(Coef)	Z	*P*-value
**Age**	203	0.0254	1.0257	0.0064	3.96	**$7.3x10^{-5}$**
**Grade**		0.6999	2.0135	0.1266	5.53	**$3.2x10^{-8}$**
**Risk allele number**		-0.0702	0.9322	0.0317	-2.22	**0.036**

f) *TERT*-only	N	Coef	Exp(Coef)	Se(Coef)	Z	*P*-value
**Age**	472	0.02981	1.03026	0.00509	5.85	**$4.8x10^{-9}$**
**Grade**		0.38720	1.47285	0.08816	4.39	**$1.1x10^{-5}$**
**Risk allele number**		-0.03874	0.96200	0.01929	-2.01	**0.045**

SUPPLEMENTARY REFERENCES

1 Louis DN, Perry A, Reifenberger G, von Deimling A, Figarella-Branger D, Cavenee WK, Ohgaki H, Wiestler OD, Kleihues P, Ellison DW (2016) The 2016 World Health Organization Classification of Tumors of the Central Nervous System: a summary. Acta Neuropathol 131: 803-820 Doi 10.1007/s00401-016-1545-1

2 Martin JS, Xu Z, Reiner AP, Mohlke KL, Sullivan P, Ren B, Hu M, Li Y (2017) HUGIn: Hi-C Unifying Genomic Interrogator. Bioinformatics: Doi 10.1093/bioinformatics/btx359
